# The transcriptional response of pathogenic *Leptospira* to peroxide reveals new defenses against infection-related oxidative stress

**DOI:** 10.1371/journal.ppat.1008904

**Published:** 2020-10-06

**Authors:** Crispin Zavala-Alvarado, Odile Sismeiro, Rachel Legendre, Hugo Varet, Giovanni Bussotti, Jan Bayram, Samuel G. Huete, Guillaume Rey, Jean-Yves Coppée, Mathieu Picardeau, Nadia Benaroudj

**Affiliations:** 1 Unité de Biologie des Spirochètes, Department of Microbiology, Institut Pasteur, Paris, France; 2 Université de Paris, Sorbonne Paris Cité, COMUE BioSPC, Paris, France; 3 Biomics Technological Plateform, Center for Technological Resources and Research, Institut Pasteur, Paris, France; 4 Bioinformatics and Biostatistics Hub, Department of Computational Biology, USR 3756 CNRS, Institut Pasteur, Paris, France; University of Montana, UNITED STATES

## Abstract

Pathogenic *Leptospira* spp. are the causative agents of the waterborne zoonotic disease leptospirosis. *Leptospira* are challenged by numerous adverse conditions, including deadly reactive oxygen species (ROS), when infecting their hosts. Withstanding ROS produced by the host innate immunity is an important strategy evolved by pathogenic *Leptospira* for persisting in and colonizing hosts. In *L*. *interrogans*, genes encoding defenses against ROS are repressed by the peroxide stress regulator, PerR. In this study, RNA sequencing was performed to characterize both the *L*. *interrogans* response to low and high concentrations of hydrogen peroxide and the PerR regulon. We showed that *Leptospira* solicit three main peroxidase machineries (catalase, cytochrome C peroxidase and peroxiredoxin) and heme to detoxify oxidants produced during peroxide stress. In addition, canonical molecular chaperones of the heat shock response and DNA repair proteins from the SOS response were required for *Leptospira* recovering from oxidative damage. Identification of the PerR regulon upon exposure to H_2_O_2_ allowed to define the contribution of this regulator in the oxidative stress response. This study has revealed a PerR-independent regulatory network involving other transcriptional regulators, two-component systems and sigma factors as well as non-coding RNAs that putatively orchestrate, in concert with PerR, the oxidative stress response. We have shown that PerR-regulated genes encoding a TonB-dependent transporter and a two-component system (VicKR) are involved in *Leptospira* tolerance to superoxide. This could represent the first defense mechanism against superoxide in *L*. *interrogans*, a bacterium lacking canonical superoxide dismutase. Our findings provide an insight into the mechanisms required by pathogenic *Leptospira* to overcome oxidative damage during infection-related conditions. This will participate in framing future hypothesis-driven studies to identify and decipher novel virulence mechanisms in this life-threatening pathogen.

## Author summary

Leptospirosis is a zoonotic infectious disease responsible for over one million of severe cases and 60 000 fatalities annually worldwide. This neglected and emerging disease has a worldwide distribution, but it mostly affects populations from developing countries in sub-tropical areas. The causative agents of leptospirosis are pathogenic bacterial *Leptospira* spp. There is a considerable deficit in our knowledge of these atypical bacteria, including their virulence mechanisms. During infection, *Leptospira* are confronted with the deadly oxidants produced by the host tissues and immune response. Here, we have identified the leptospiral factors necessary for overcoming infection-related oxidative stress. We found that *Leptospira* solicit peroxidases to detoxify oxidants as well as chaperones of the heat shock response and DNA repair proteins of the SOS response to recover from oxidative damage. Moreover, our study indicates that the oxidative stress response is orchestrated by a regulatory network involving PerR and other transcriptional regulators, sigma factors, two component systems, and putative non-coding RNAs. These findings provide insights into the mechanisms required by pathogenic *Leptospira* to tolerate infection-related oxidants and could help identifying novel virulence factors and developing new therapeutic targets.

## Introduction

In order to invade a host and establish persistent colonization, pathogens have evolved a variety of strategies to resist, circumvent, or counteract host defenses. Pathogens synthesize enzymes or molecules to eliminate host-produced bactericidal compounds, secrete effectors inhibiting or subverting the host innate immunity, or form biofilms enabling resistance to host defenses.

The strategies used by pathogenic *Leptospira* for successful host colonization and virulence are not fully understood. These aerobic Gram-negative bacteria of the spirochetal phylum are the causative agents of leptospirosis, a widespread zoonosis [1]. Although recognized as a health threat among impoverished populations in developing countries and tropical areas [2], reported cases of leptospirosis are also on the rise in developed countries under temperate climates [3]. Rodents are the main reservoir for leptospires as the bacteria asymptomatically colonize the proximal renal tubules of these mammals. Infected animals shed bacteria in the environment by their urine and leptospires are transmitted to other animals and humans mostly by exposure to contaminated soils and water. *Leptospira* penetrate mucous membranes or abraded skin, enter the bloodstream and rapidly disseminate to multiple tissues and organs including kidney, liver and lungs. Clinical manifestations range from a mild flu-like febrile state to more severe and fatal cases leading to hemorrhages and multiple organ failure. The lack of efficient tools and techniques for genetic manipulation of *Leptospira* spp. and their fastidious growth in laboratory conditions have greatly hampered and limited our understanding of their mechanisms of pathogenicity and virulence [4,5].

As part of the host innate immunity response, reactive oxygen species (ROS), *i*.*e*. superoxide anion (^•^O_2_^-^), hydrogen peroxide, (H_2_O_2_), hydroxyl radicals (^•^OH), hypochlorous acid (HOCl), and nitric oxide anion (^•^NO) are produced upon infection by *Leptospira*. Indeed, the internalization of pathogenic *Leptospira* by macrophages and concomitant production of these oxidants have been demonstrated *in vitro* [6], and leptospirosis-associated oxidative stress has been observed in leptospirosis patients [7] and infected animals [8]. Consistent with these findings was the demonstration that catalase, which catalyzes the degradation of H_2_O_2_, is required for *Leptospira interrogans* virulence [9].

Pathogenic *Leptospira* spp. are among the rare examples of Gram-negative bacteria in which defenses against peroxide stress, such as catalase, are controlled by a peroxide stress regulator (PerR) and not by OxyR [10]. PerR is a peroxide-sensing transcriptional repressor that belongs to the Fur (Ferric uptake regulator) family of regulators, mostly present in Gram-positive bacteria [11]. The *B*. *subtilis* PerR is in a DNA-binding prone conformation in the presence of a regulatory metal (Fe^2+^) [12]. Upon oxidation by H_2_O_2_, PerR releases its regulatory metal and switches to a conformation that cannot bind DNA, leading to the alleviation of gene repression [13,14].

We have conducted a structural and functional characterization of PerR in *L*. *interrogans* and showed that *Leptospira* PerR exhibits the typical metal-induced conformational switch controlling DNA binding and release [15]. Our findings indicated that not only does *Leptospira* PerR repress defenses against H_2_O_2_, but a *perR* mutant also had a decreased fitness in other host-related stress conditions including in the presence of superoxide [15]. Interestingly, it was shown that *perR* is up-regulated when *Leptospira* are exposed *in vitro* to hydrogen peroxide [15] as well as when *Leptospira* are cultivated *in vivo* using Dialysis Membrane Chambers (DMCs) in rats [16], which strongly suggests a role of PerR in the adaptation of pathogenic *Leptospira* to a mammalian host.

In order to identify the mechanisms solicited by pathogenic *Leptospira* to adapt to oxidative stress, we determined the global transcriptional response of *L*. *interrogans* to H_2_O_2_ and assessed the role of PerR in this response. This has revealed the leptospiral factors constituting the first-line of defense against the ROS that *Leptospira* might encounter when infecting a mammalian host. In addition, our study has identified repair mechanisms allowing leptospires to recover from oxidative damage. Putative regulatory non-coding RNAs were also pinpointed, indicating the complexity of the regulatory network controlling the response to peroxide. We have also identified novel PerR-regulated factors involved in *Leptospira* survival in the presence of superoxide and assessed their role in *Leptospira* virulence.

## Results

### *Leptospira* transcriptional response to a sublethal concentration of hydrogen peroxide

In order to characterize the transcriptional response of pathogenic *Leptospira* to hydrogen peroxide, we exposed exponentially growing *L*. *interrogans* cells to a sublethal concentration of this oxidant. A 30 min treatment with 10 μM H_2_O_2_ (in the presence of iron) was chosen during pilot experiments as having no significant effect on *Leptospira* viability and growth during logarithmic phase while increasing expression of H_2_O_2_-responsive genes such as *perR* [15]. RNA-Seq (RNA sequencing) was performed to assess RNA abundance and comparison with untreated cells identified a total of 21 genes with differential transcript abundance (see S1 Table for complete data set). Among those, only 13 and 1 genes were respectively up- and down-regulated by at least two-fold with p-values ≤0.05 (See Table 1).

**Table 1:** Differentially expressed genes upon exposure to sublethal dose of H_2_O_2_.

ORF ID^a^	Gene	Function	COGs^b^	Log_2_FC	Adjusted p-value	FC (RT-qPCR)^c^
***Up-regulated genes***						
LIMLP_02795 (LIC12927/LA0666)	*ccp*	Cytochrome C peroxidase	P	4.764*	5.31e-43	38.900
LIMLP_05955 (LIC11219/LA2809)	*ahpC*	Peroxiredoxin/alkylperoxiredoxin reductase	O	3.145*	3.63e-20	11.742
LIMLP_05960 (LIC11220/LA2808)	*sufB*	Fe-S cluster assembly protein	O	1.056*	1.60e-08	1.880
LIMLP_10145 (LIC12032/LA1859)	*katE*	Catalase	P	1.786	2.11e-08	3.477
LIMLP_10150 (LIC12033/LA1858)		Ankyrin repeat-containing protein	S	2.051*	2.30e-11	4.183
LIMLP_10155 (LIC12034/LA1857)	*perR*	Regulator Fur familly	T	2.319*	1.02e-39	6.827
LIMLP_17840 (LIC20008/LB010)	*hemA*	Glutamyl-tRNA reductase	H	1.771*	1.16e-10	3.389
LIMLP_17845 (LIC20009/ LB011)	*hemC/D*	Porphobilinogen deaminase	H	1.617*	6.57e-13	2.328
LIMLP_17850 (LIC20010/ LB012)	*hemB*	Delta-aminolevulinic acid dehydratase	H	1.455*	2.65e-14	2.064
LIMLP_17855 (LIC20011/ LB013)	*hemL*	Glutamate-1 semialdehyde aminotransferase	H	1.262*	1.19e-07	2.193
LIMLP_17860 (LIC20012/ LB014)		Signal transduction histidine kinase	T	1.035*	2.67e-03	2.470
LIMLP_17865 (LIC20013/ LB015)		Response regulator CheY	K	1.166*	1.01e-03	2.012
LIMLP_17870 (LIC20014/ LB016)	*hemE*	Uroporphyrinogen decarboxylase	H	1.033*	1.77e-02	2.059
***Down-regulated genes***						
LIMLP_18600 (LIC20149/ LB187)		Permease of the Major facilitator superfamily	P	-1.001	1.17e-04	0.894

Significantly up-and down-regulated genes upon a 30 min exposure to 10 μM H_2_O_2_ with a Log_2_FC cutoff of ± 1 and an adjusted p-value cutoff of 0.05.

^a^ Gene numeration is according to Satou *et al*. [17]. Corresponding genes of *L*. *interrogans* serovar lai strain 56601 and serovar Copenhageni strain Fiocruz L1-130 are indicated in parenthesis.

^b^ The COG functional categories are H, coenzyme transport and metabolism; K, transcription; O, posttranslational modification, protein turnover, chaperones; P, inorganic ion transport and metabolism; S, function unknown; T, signal transduction and metabolism.

^c^ Fold change in gene expression upon a 30 min exposure to 10 μM H_2_O_2_ obtained by RT-qPCR experiments.

**Fig 1:**
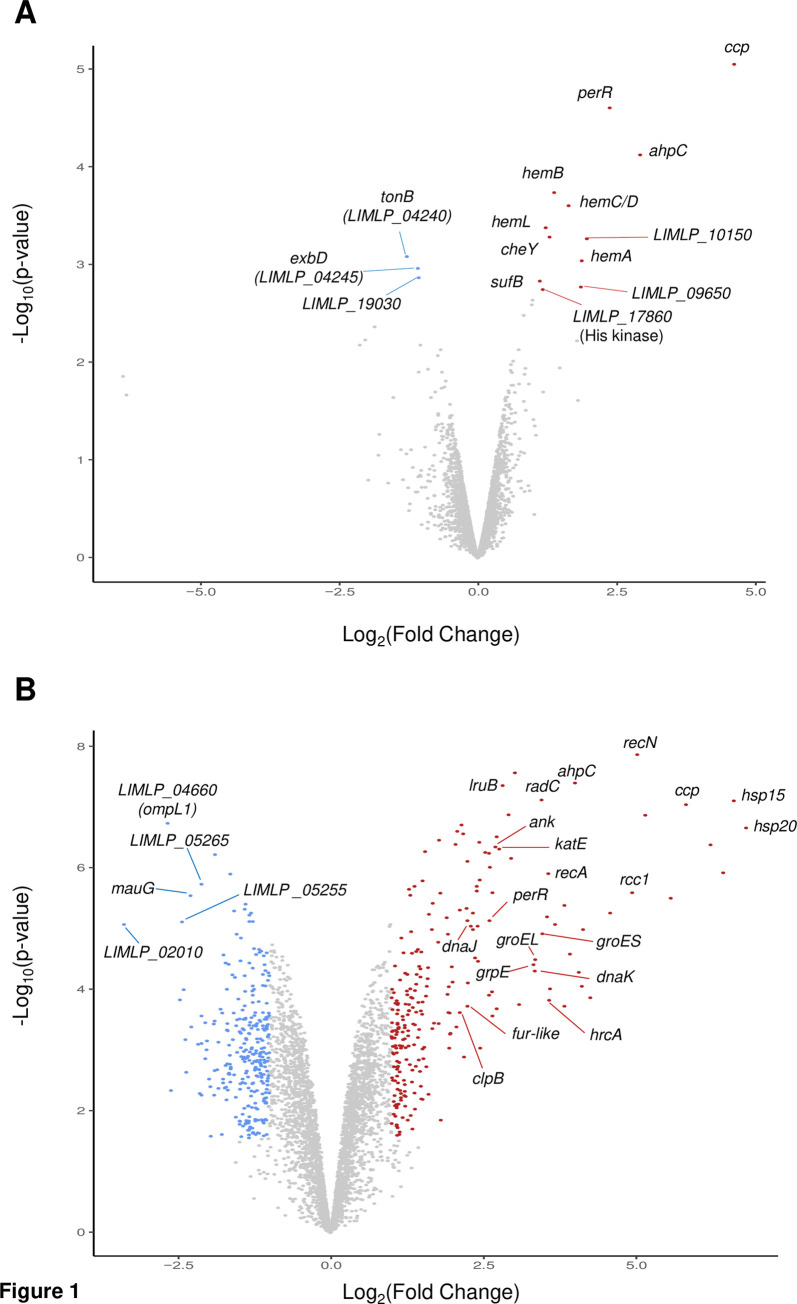
Volcano representation of differentially-expressed genes upon exposure to hydrogen peroxide. Up- and down-regulated genes upon a 30 min exposure to 10 μM H_2_O_2_ (A) or 1 hour exposure to 1 mM H_2_O_2_ (B) were graphically represented by a Volcano analysis. Red and blue dots indicate up- and down-regulated genes, respectively, with significant change in expression with a Log_2_FC cutoff of ±1 and p-value<0.05. Representative genes are labeled.

* Genes significantly up-and down-regulated by Volcano analysis (Log_2_FC cutoff of 1 and p-value cutoff of 0.05 as seen in Fig 1A).

Under a low concentration of H_2_O_2_, LIMLP_10145, encoding a catalase, and LIMLP_02795 and LIMLP_05955, coding respectively for a cytochrome C peroxidase and for a peroxiredoxin, were up-regulated with a Log_2_FC (Log_2_ Fold Change) of 1.79, 4.76 and 3.14 respectively.

The catalase encoded by LIMLP_10145 (*katE*) is a monofunctional heme-containing hydroperoxidase, whose catalase activity and periplasmic localization were experimentally demonstrated in pathogenic *Leptospira* [9,18,19]. The immediate upstream ORF (LIMLP_10150), encoding an ankyrin repeat-containing protein, was also up-regulated with a comparable fold value. In bacteria such as *Pseudomonas aeruginosa* and *Campylobacter jejuni*, a protein with ankyrin repeats were found to be required for catalase activity, probably by allowing heme binding [20,21]. In *L*. *interrogans*, *katE* and *ank* were organized as an operon (S1 Fig) and significant up-regulation of the *ank-katE* operon upon exposure to sublethal dose of H_2_O_2_ was confirmed by RT-qPCR (Table 1 and S1 Fig).

The significantly up-regulated *ahpC* gene (LIMLP_05955) encodes a peroxiredoxin that reduces H_2_O_2_ and *tert*-Butyl hydroxyperoxide [22]. The SufB-encoding LIMLP_05960 located in the vicinity of *ahpC* was also up-regulated with a 2-fold. *SufB* encodes a polypeptide involved in Fe-S cluster assembly proteins. In bacteria such as *Escherichia coli*, SufB is part of a complex composed of SufB, SufD and the SufC ATPase. *SufB* is normally found in an operon with *sufC* and *sufD* as well as with the other factors of the Suf machinery, i. e. *sufE* and *sufS*. *L*. *interrogans* genome contains a putative *suf* cluster (LIMLP_14560–14580 ORFs) and SufE (encoded by LIMLP_05090) but none of the other *suf* ORFs were significantly regulated by sublethal dose of H_2_O_2_. The SufB-encoding LIMLP_05960 shares 40% and 47% identity with SufB from *E*. *coli* and *B*. *subtilis*, respectively, and most importantly it does contain the critical cysteine residue suggesting that the isolated LIMLP_05960 encodes a *bona fide* SufB participating in the SufBC_2_D complex of Fe-S cluster biogenesis. A function or cooperation of SufB with AhpC in H_2_O_2_ detoxification remains to be demonstrated.

LIMLP_02795 was another peroxidase-encoding ORF that was greatly up-regulated in the presence of H_2_O_2_. LIMLP_02795 encodes a putative Cytochrome C Peroxidase (CCP) that catalyzes the reduction of H_2_O_2_ into H_2_O using the ferrocytochrome as an electron donor. In several *L*. *interrogans* genomes, this ORF is annotated as a MauG, a class of Cytochrome C Peroxidase that catalyzes the oxidation of methylamine dehydrogenase (MADH) into tryptophan tryptophylquinone (TTQ) in the methylamine metabolism pathway. LIMLP_02795 exhibits two heme domains with the conventional heme binding motif CXXCH that exists in both CCP and MauG proteins, but it lacks the tyrosine axial ligand for heme (Tyr294 in *Paracoccus denitrificans*, [23]) that is conserved in all MauGs but replaced by a methionine or histidine residue in CCPs. Therefore, it is very likely that LIMLP_02795 encodes a CCP with a peroxidase activity that is not involved in the methylamine metabolism pathway.

In addition to these three peroxidases, whose increased expression was confirmed by RT-qPCR (Table 1 and S1 and S2 Figs), several ORFs encoding components of heme biosynthesis (LIMLP_17840–17865) were up-regulated by 2 to 3.4-fold (Table 1). *Leptospira*, unlike other spirochetes, possess a complete heme biosynthesis functional pathway [24]. The ORFs encoding the glutamyl-tRNA reductase (*hemA*), porphobilinogen deaminase (*hemC/D*), delta-aminolevulinic acid dehydratase (*hemB*), glutamate-1 semialdehyde aminotransferase (*hemL*), uroporphyrinogen-III decarboxylase (*hemE*), coproporphyrinogen-III oxidase (*hemN/F*), as well as a two-component system (TCS) (LIMLP_17860 and LIMLP_17865) were organized as an operon (S3 Fig). RT-qPCR confirmed the significance of the up-regulation of *hemA*, *hemC/D*, *hemL*, and of the LIMLP_17860-encoded histidine kinase of the TCS (Table 1 and S3 Fig).

When pathogenic *Leptospira* cells are exposed to 10 μM H_2_O_2_, the only ORF that was down-regulated was that encoding a permease (LIMLP_18600; with a Log_2_FC of -1). This permease is a putative Major Facilitator Superfamily (MFS) transporter and is predicted to contain 12 transmembrane helixes. This permease-encoding ORF is the second gene of a bicistronic operon where a heme oxygenase-encoding ORF (LIMLP_18595) is the first (S4 Fig). Expression of the heme oxygenase ORF was not significantly changed by the exposure to 10 μM H_2_O_2_ (S4 Fig and S1 Table).

Plotting statistical significance in function of fold change confirmed that *katE*, *ccp*, *ahpC*, *perR*, and several genes of the heme biosynthesis pathway were among the genes the expression of which was significantly up-regulated (Fig 1A).

Notably, after a 2-hour exposure of *L*. *interrogans* to 10 μM H_2_O_2_, the expression of the peroxidases and heme biosynthesis genes returns to a level closer to that observed in the absence of H_2_O_2_ (S1–S4 Figs). Altogether, these data indicate that pathogenic *Leptospira* respond to a sublethal dose of H_2_O_2_ by soliciting three peroxidases and heme, and that the up-regulated peroxidase and catalase activities are probably sufficient to degrade H_2_O_2_ and allow survival of *Leptospira* in the conditions tested in this study.

### *Leptospira* transcriptional response to lethal concentration of hydrogen peroxide

In order to better reproduce harmful oxidative stress encountered during infection, we performed similar RNA-Seq experiments upon 1-hour exposure to 1 mM H_2_O_2_. In this condition, *Leptospira* survival was 60% ± 2.735 as assessed by plating on EMJH agar plates. Comparison with untreated cells identified a total of 2145 genes with differential transcript abundance (see S2 Table for complete data set). Among those, 243 and 296 genes were respectively up- and down-regulated by ≥2.0 fold with p-values ≤0.05. The volcano representation exhibited more scattered data points (Fig 1B), bearing witness to a higher number of genes with significantly and statistically changed expression than when *Leptospira* are exposed to sublethal dose of H_2_O_2_ (Fig 1A).

**Fig 2:**
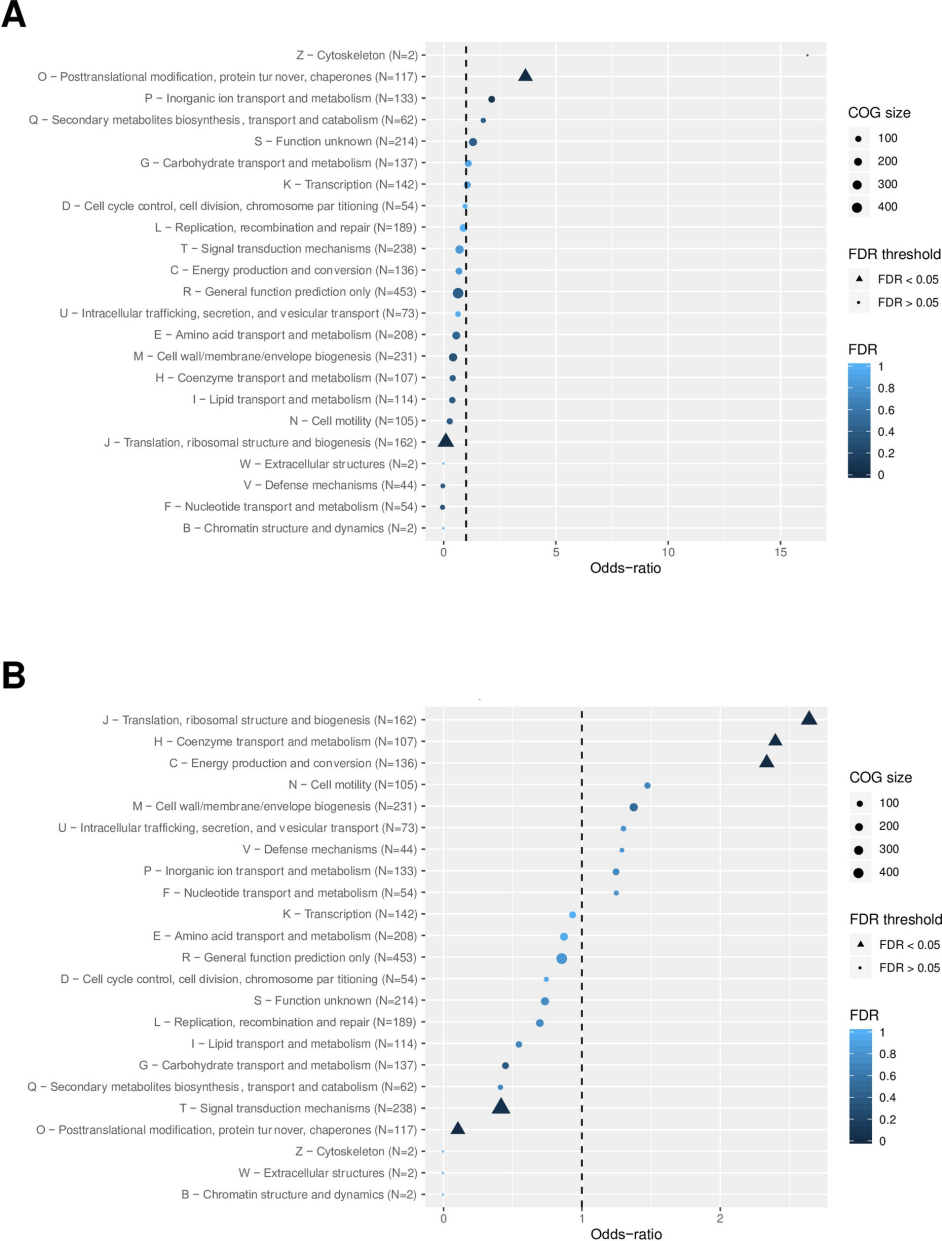
Classification of differentially-expressed genes upon exposure to lethal dose of hydrogen peroxide. ORFs with significantly changed expression when *L*. *interrogans* were exposed to 1 mM H_2_O_2_ for 1h were classified according to the COG (Clusters of Orthologous Groups). A Log_2_FC cutoff of ±1 were applied for the up-regulated (A) and down-regulated (B) ORFs, respectively, with an adjusted p-value<0.005. An odd-ratio higher or lower than 1 (dashed line) indicates an over- or under-representation of a functional category, respectively, and a COG category with a False Discovery Rate (FDR) lower than 5% is considered as enriched. The functional categories are indicated on the left.

Differentially expressed genes were classified into COG functional categories and the obtained COG frequencies were compared to the frequency of the genes in the genome. As seen in Fig 2, the up-regulated genes were enriched in the post-translational modification, protein turnover, and chaperones categories whereas down-regulated genes mainly fell into metabolism, translation and ribosomal structure and biogenesis, coenzyme transport and metabolism, and energy production and conversion categories.

**Fig 3:**
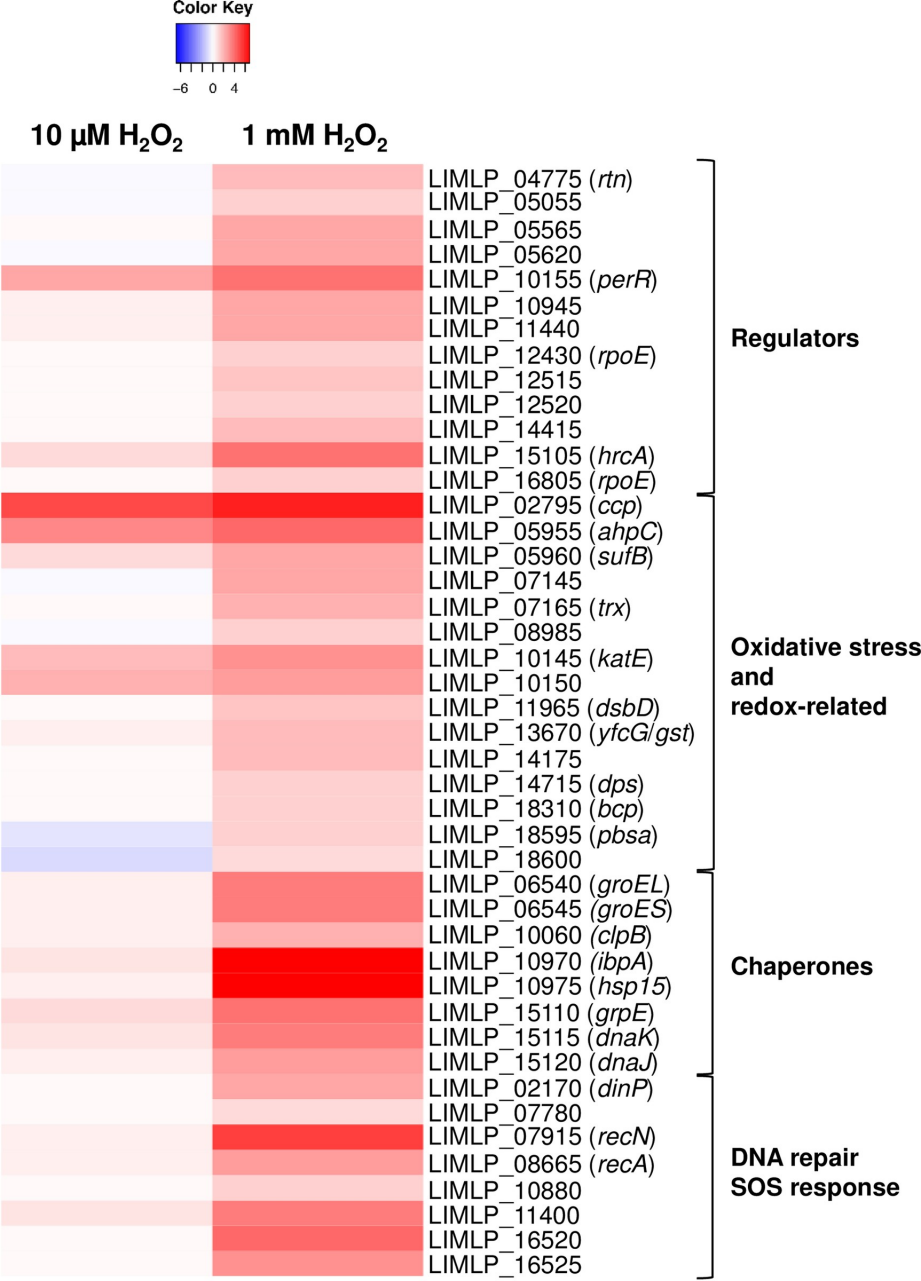
Comparison of up-regulated genes upon exposure to sublethal and lethal doses of hydrogen peroxide. The differential expression of selected up-regulated genes determined by RNA-Seq when *L*. *interrogans* are exposed 1 hour to 1 mM H_2_O_2_ was compared to that of *L*. *interrogans* exposed 30 min to 10 μM H_2_O_2_. Differential expression in H_2_O_2_-treated *Leptospira* was obtained by normalization with untreated *Leptospira*. Genes are organized by their function and their number and name are indicated on the right. The Heat Map color from blue to red indicates low to high Log_2_FC.

As in the presence of low dose of H_2_O_2_, the *ank-katE* operon (LIMLP_10150–10145), *ccp* (LIMLP_02795) and *ahpC* (LIMLP_05955) were up-regulated in the presence of 1 mM H_2_O_2_ but with higher fold changes (with Log_2_FC values of 2.7, 5.8 and 4, respectively, see Fig 3 and S3 Table). The ORF upstream *ahpC* that encodes a SufB (LIMLP_05960) was also significantly up-regulated (with a with Log_2_FC value of 2.2). Likewise, *perR* expression was greater in the presence of 1 mM H_2_O_2_ (with Log_2_FC value of 3.5, see Fig 3 and S3 Table). All these up-regulations were confirmed by RT-qPCR experiments (S3 Table).

Additional ORFs encoding factors related to oxidative stress and redox maintenance were also up-regulated (Fig 3 and S2 and S3 Tables). An ORF encoding a thiol oxidoreductase (LIMLP_07145) exhibiting two cytochrome C-like (heme binding) domains was up-regulated with a Log_2_FC value of 2.2. LIMLP_07145 was located immediately downstream an ORF (LIMLP_07150) encoding a protein with five chromosome condensation regulator (RCC1) domains that was up-regulated with a Log_2_FC value of about 5. LIMLP_07145–07150 are probably organized as a bicistronic operon as predicted in Zhukova *et al*. [25]. A second thiol peroxidase-encoding ORF (LIMLP_14175) exhibiting a single cytochrome C-like domain was also up-regulated (Log_2_FC value of 1.8). This ORF might be part of the operon LIMLP_14170–14180 where LIMLP_14170 and LIMP_14180, two ORFs annotated as Imelysins (iron-regulated proteins), were also up-regulated (Log_2_FC value of 2.8 and 1.4, respectively). Of note, the Imelysin encoded by LIMLP_14170 is the LruB protein that was shown to be associated with *Leptospira*-induced uveitis [26].

A thioredoxin disulfide reductase (encoded by LIMLP_07165) was up-regulated (Log_2_FC value of 1.9, see Fig 3 and S3 Table). This protein has been shown to catalyze *in vitro* the NADPH-dependent reduction of a thioredoxin encoded by LIMLP_09870 [27]. The LIMLP_09870 was only slightly up-regulated in the presence of 1 mM H_2_O_2_ (Log_2_FC value of 0.8, see S2 Table).

Other thiol peroxidase-encoding ORFs were up-regulated, including LIMLP_08985 that encodes a glutaredoxin, LIMLP_11965 that codes for the periplasmic thiol disulfide interchange protein DsbD, and LIMLP_18310 that encodes a bacterioferritin comigratory protein (Bcp) (See Fig 3 and S3 Table). An ORF encoding a putative Glutathione S transferase (LIMLP_13670) had an increased expression in the presence of 1 mM H_2_O_2_ (Log_2_FC value of 1.76), as did an ORF annotated as DNA binding stress protein (Dps) (Log_2_FC value of 1.09) (See Fig 3 and S3 Table).

Major pathways involved in repair of damaged cellular components were dramatically up-regulated when *Leptospira* were exposed to a lethal dose of H_2_O_2_. Indeed, several genes encoding molecular chaperones had an increased expression in the presence of 1 mM H_2_O_2_ (Fig 3 and S3 Table). Two ORFs encoding small heat shock proteins (sHSP), probably organized as a bicistronic operon (LIMLP_10970–10975), exhibited a significant increase in expression (Log_2_FC values of about 6). The LIMLP_15105–15120 cluster encoding the DnaK/DnaJ/GrpE molecular chaperone machinery and its putative repressor HrcA, was significantly up-regulated with Log_2_FC values of 2.6–3.6. Similarly, the GroES-GroEL operon (encoded by LIMLP_06545–06540) was up-regulated with a Log_2_FC value of 3.3. The *clpB* gene (LIMLP_10060) also had an increased expression (Log_2_FC value of 2.1). Thus, the machinery necessary for preventing protein aggregation and promoting protein refolding is solicited when *Leptospira* are exposed to high doses of H_2_O_2_.

Genes encoding several components of the SOS response, a regulatory network stimulated by DNA damage-inducing stress, had a higher expression in the presence of 1 mM H_2_O_2_ (Fig 3 and S3 Table). Indeed, ORFs encoding the recombinase A (*recA*, LIMLP_08665), the DNA repair protein RecN (LIMLP_07915), the DNA polymerase IV (*dinP*, LIMLP_02170) as well as the repressor of the SOS response LexA1 (LIMLP_11440) were significantly up-regulated. Other factors putatively involved in DNA repair but not under the control of LexA1 [28,29] had also an increased expression, including the DNA mismatch repair protein MutS (LIMLP_07780, Log_2_FC value of 1) and the DNA repair protein RadC (LIMLP_11400, Log_2_FC value of 3.4).

One remarkably up-regulated ORF (LIMLP_00895) was located into a genomic region previously identified as an island enriched in prophage genes ranging from LIMLP_00855 to LIMLP_01005 and referred to as prophage 1 [29,30] (S3 Table). Also, another cluster enriched in prophage genes (from LIMLP_13010 to LIMLP_13095), referred to as prophage 2 [29], contains 4 ORFs (LIMLP_13010, LIMLP_13015, LIMLP_13020, and LIMLP_13025) that were up-regulated in the presence of 1 mM H_2_O_2_ (S3 Table).

Down-regulated genes were mainly genes putatively involved in translation and metabolism (Fig 2B and S4 Table). 14 ORFs encoding ribosomal proteins, a translation initiation factor (LIMLP_03190), a ribosome maturation factor (LIMLP_07600), a RNA polymerase RpoA (LIMLP_03215), and a transcription termination factor RhoA (LIMLP_13190) were among them.

A cluster of genes encoding the ATP synthase complex (LIMLP_06050–06080) was down-regulated, indicating that *Leptospira* decrease ATP synthesis upon exposure to high dose of H_2_O_2_ (S4 Table). Another metabolic pathway that was down-regulated in this condition was the cobalamin (vitamin B12) biosynthesis pathway. Indeed, 15 out 17 genes of the cobI/III cluster (LIMLP_18460–18530) were significantly down-regulated (S4 Table).

A cluster of four genes encoding proteins of the CRISPR-Cas machinery (*csh2*, LIMLP_2870; *cas8*, LIMLP_2875; *cas5*, LIMLP_2880; *cas3*, LIMLP_2880) putatively involved in phage defense were down-regulated (see S4 Table).

Finally, several genes related to motility/chemotaxis were down-regulated when *Leptospira* are exposed to a high dose of H_2_O_2_. Several of these genes encode constituents of the endoflagellum basal body (*flgGAHIJ*, LIMLP_06485–06505), of the flagellar export apparatus (*fliOPQR-FlhBA*, LIMLP_06690–06715; *fliL*, LIMLP_14615 and LIMLP_14620), and of the flagellar motor stator (*motAB*, LIMLP14625-14630), and chemotaxis-related proteins (*cheBDW*-*mcp*, LIMLP_07420–07435) (see S2 Table).

### Contribution of PerR in the pathogenic *Leptospira* response to peroxide stress

Comparison of the transcriptome of a *perR* mutant with that of WT strain allowed determination of the PerR regulon in *L*. *interrogans*. In the *perR* mutant, 5 and 12 ORFs were up- and down-regulated, respectively, with a log_2_FC cutoff of 1 and a p-value below 0.05 (Table 2 and S1 Table).

**Table 2:** Differentially expressed genes upon *perR* inactivation.

ORF ID^a^	Gene	Function	Log_2_FC	Adjusted p-value	FC (RT-qPCR)^b^
***Down-regulated genes***					
LIMLP_04090 (LIC12679/LA0980)	*thic*	Thiamine biosynthesis protein	-2.073	3.72e-02	
LIMLP_04240 (LIC10889/LA3247)	*tonb*	Energy transducer TonB	-4.601	2.03e-13	0.00722
LIMLP_04245 (LIC10890/LA3246)	*exbD*	Biopolymer transport protein ExbD/TolR	-4.606	7.90e-13	0.00737
LIMLP_04250 (LIC10891/LA3245)	*exbD*	Biopolymer transport protein ExbD/TolR	-5.355	4.93e-15	0.00128
LIMLP_04255 (LIC10892/LA3244)	*exbB*	Biopolymer transport protein ExbB/TolQ	-5.478	3.00e-22	0.00193
LIMLP_04260 (LIC10893/LA3243)		Hypothetical	-1.519	4.27e-02	1.261
LIMLP_04270 (LIC10895-96/LA3242)		TonB-dependent receptor	-3.262	2.32e-05	0.0355
LIMLP_04275 (LIC10897/LA3241)		Hypothetical	-3.888	4.42e-05	0.00918
LIMLP_04280 (LIC10898/LA3240)	*lipl48*	Hypothetical	-5.506	2.03e-13	0.00372
LIMLP_09650 (LIC11935/LA1968)*		Hypothetical	-1.787	3.26e-02	
LIMLP_15470 (LIC10454/LA3793)		Putative hemolysin	-2.154	3.32e-12	0.3041
LIMLP_16720 (LIC13269/LA4102)	*vicR*	Response regulator	-1.611	5.80e-07	0.0752
***Up-regulated genes***					
LIMLP_02010 (LIC13086/LA3867)**		Hypothetical lipoprotein	1.029	4.08e-02	
LIMLP_02795 (LIC12927/LA0666)*	*ccp*	Cytochrome C peroxidase	2.773	8.69e-18	7.943
LIMLP_05955 (LIC11219/LA2809)*	*ahpC*	Peroxiredoxin/alkylperoxiredoxin reductase	1.539	1.23e-05	2.01
LIMLP_10145 (LIC12032/LA1859)*	*katE*	Catalase	2.637	2.59e-24	4.897
LIMLP_10150 (LIC12033/LA1858)*		Ankyrin repeat-containing protein	2.867	4.65e-29	5.783

Significantly up-and down-regulated genes in the *perR* mutant with a Log_2_FC cutoff of ± 1 and an adjusted p-value cutoff of 0.05.

^a^ Gene numeration is according to Satou et al. [17]. Corresponding genes of *L*. *interrogans* serovar lai strain 56601 and serovar Copenhageni strain Fiocruz L1-130 are indicated in parenthesis.

^b^ Fold change in gene expression upon *perR* inactivation obtained by RT-qPCR experiments.

* ORFs significantly up-regulated upon exposure to H_2_O_2_ (Log_2_FC cutoff of -1, adjusted p-value cutoff of 0.05).

** ORFs significantly down-regulated upon exposure to H_2_O_2_ (Log_2_FC cutoff of -1, adjusted p-value cutoff of 0.05).

**Fig 4:**
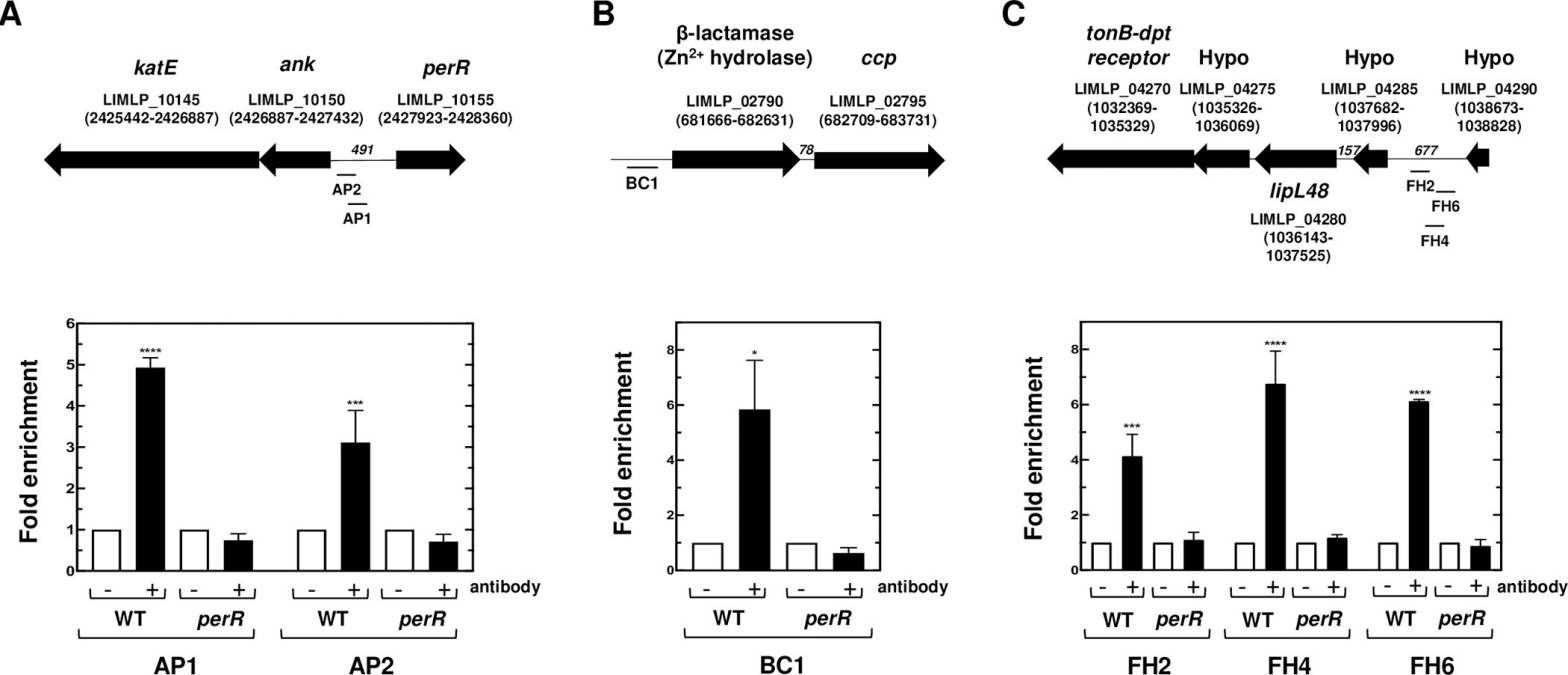
*In vivo* interaction between PerR and promoter regions of PerR-controlled genes. Chromatin immunoprecipitation was performed on *L*. *interrogans* WT and *perR* (M776) mutant strains in the presence or absence of the anti-PerR antibody as described in the Material and Methods section. Co-immunoprecipitated DNA fragments located in the *ank-katE* operon locus (A), in the *ccp* locus (B) and in the locus encoding a TonB-dependent transport system (C) were amplified by qPCR. The location of amplified fragments is indicated below the schematic representation of their respective locus. ORF location in the genome (according to Satou *et al*. [17]) is indicated into parenthesis and the number of nucleotides between different ORFs is indicated in italic. Data are represented as fold enrichments and are means and SD of two independent biological replicates (****, adjusted p-value<0.0001; ***, adjusted p-value of 0.0006; *, adjusted p-value of 0.0422).

The *ank-katE* operon, encoded by LIMLP_10150–10145, *ahpC*, encoded by LIMLP_05955, and *ccp*, encoded by LIMLP_02795, were up-regulated upon *perR* inactivation. *KatE*, *ahpC* and *ccp* up-regulation is consistent with the high resistance of the *perR* mutant to H_2_O_2_ concentrations that are otherwise lethal for the WT strain [10,15]. ChIP-PCR experiments showed that when *Leptospira* were cultivated in EMJH medium, PerR was bound to DNA fragments comprising the 25 to 191 bp upstream region to the *ank-katE* operon (Fig 4A). Lower non-significant PerR binding was detected inside the *ank-katE* operon (S5 Fig). This is consistent with a direct repression of the *ank-katE* operon by PerR. Significant PerR binding was observed from 150 to 350 bp upstream the LIMLP_02790, the ORF located immediately upstream the *ccp* ORF (Fig 4B and S5 Fig). ChIP experiments also showed a rather weak binding upstream the *ahpC* ORF (-298 to -123 region) (S5 Fig).

Identification of the PerR regulon has allowed the identification of genes whose expression is activated directly or indirectly by PerR (Table 2). A cluster composed of genes encoding a TonB-dependent transport (TBDT) system (LIMLP_04240–04255, encoding TonB, two ExbDs and ExbB, respectively) was dramatically down-regulated in the *perR* mutant. The downstream TonB-dependent receptor- and LipL48-encoding ORFs were also down-regulated upon *perR* inactivation. LIMLP_04240 (*tonB*), LIMLP_04245 (*exbD*), and LIMLP_04250 (*exbD*) were organized as an operon and LIMLP_04270 (encoding the TonB-dependent receptor), LIMLP_04275, LIMLP_04280 (*lipl48*) and LIMLP_04285 constituted another operon (S6 Fig). ChIP-PCR assays indicated that a region upstream the LIMLP_04285–04270 operon (mapped from 436 to 617 bp) was significantly bound by PerR (Fig 4C) whereas lower binding was detected upstream the LIMLP_04265 ORF and within the LIMLP_04245 (S7 Fig). A bicistronic operon composed of the response regulator VicR (LIMLP_16720) and the histidine kinase VicK (LIMLP_16725) of a two-component system (S8 Fig) was also down-regulated (with a Log_2_FC of -1.61 and -0.91, respectively) (Table 2 and S1 Table).

Interestingly, among the PerR regulon, several genes whose expression is repressed by PerR were up-regulated when *Leptospira* were exposed to H_2_O_2_. Indeed, the expression of the *ank-katE* operon, *ahpC* and *ccp* were up-regulated in the *perR* mutant and in the presence of H_2_O_2_ whereas the expression of the ORFs encoding TonB, ExbD, ExbB, the TonB-dependent receptor, LipL48, VicK and VicR was not dramatically or significantly altered by the presence of H_2_O_2_.

**Fig 5:**
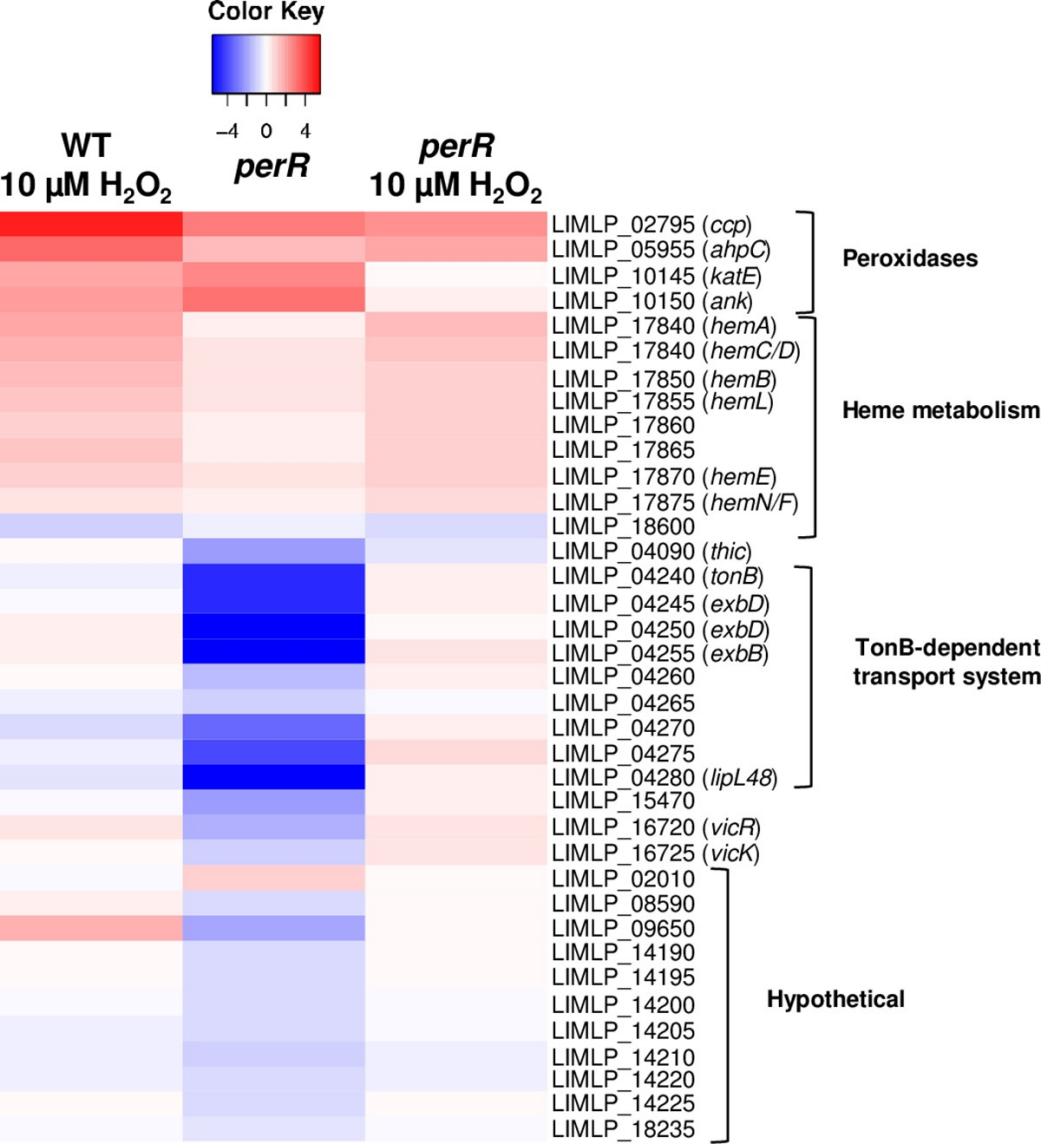
Comparison of differentially-expressed genes upon exposure to hydrogen peroxide and *perR* inactivation. The expression of selected differentially-expressed genes determined by RNA-Seq when WT *L*. *interrogans* are exposed 30 min to 10 μM H_2_O_2_ was compared to that in the *perR* (M776) mutant in the absence or presence of 10 μM H_2_O_2_. Gene expression in the *perR* mutant strain with H_2_O_2_ was normalized here with that of the *perR* mutant strain without H_2_O_2_. Genes are organized by their function and their number and name are indicated on the right. The Heat Map color from blue to red indicates low to high Log_2_FC.

In order to determine the exact contribution of PerR in the gene expression increase upon exposure to H_2_O_2_ in *Leptospira*, the transcriptome of the *perR* mutant exposed to a sublethal dose of H_2_O_2_ was also obtained (see S1 Table for a complete set of data). The *ank-katE* operon, whose expression is directly repressed by PerR and increased in the presence of H_2_O_2_ in WT *Leptospira*, was not up-regulated in the presence of H_2_O_2_ when *perR* was inactivated (Fig 5 and S1 Table). The amount of *ank-katE* operon expression in the *perR* mutant is in fact comparable to that in WT *Leptospira* exposed to a deadly dose of H_2_O_2_. This indicates that derepression of the *ank-katE* operon induced by the presence of H_2_O_2_ probably solely reflects PerR dissociation from DNA when PerR is oxidized. *AhpC* and *ccp* were still significantly up-regulated in the presence of H_2_O_2_ in the *perR* mutant (with Log_2_FC values of 2.30 and 1.88, respectively, see Fig 5 and S1 Table). Therefore, an H_2_O_2_-induced mechanism increases the expression of these two genes even in the absence of PerR, even though their expression is repressed by this regulator.

The expression of heme biosynthesis genes was not under the control of PerR and, as expected, their expression was still up-regulated in the *perR* mutant in the presence of H_2_O_2_ (Fig 5 and S1 Table).

Altogether, these findings indicate that not all H_2_O_2_-regulated genes belong to the PerR regulon in pathogenic *Leptospira* and several PerR-regulated genes were not regulated by H_2_O_2_ (Fig 5).

### Identification of differentially expressed non-coding RNAs in the presence of hydrogen peroxide

In order to identify non-coding RNAs (ncRNAs) whose expression is changed in the presence of hydrogen peroxide, non-coding genome regions of RNA-Seq data were also analyzed. When *Leptospira* were exposed to 10 μM H_2_O_2_ for 30 min, only 19 ncRNAs were differentially expressed (see S5 Table and S6 Table for the complete set of data). The most highly up-regulated ncRNAs were rh859, rh3130 and rh3999 (S5 Table). When *Leptospira* were exposed to a lethal dose of hydrogen peroxide (1 mM H_2_O_2_ for 1h), a higher number of differentially expressed ncRNAs was detected. Indeed, 416 and 102 ncRNAs were up- and down-regulated, respectively (S6 Table). 63 ncRNAs were up-regulated with a Log_2_FC above 1. Rh3130 and rh3352 were the two most highly up-regulated ncRNAs with Log_2_FC above 7 and rh288 was up-regulated with a Log_2_FC of 3.81 (Table 3). 53 ncRNAs were down-regulated with a Log_2_FC below -1. Rh967 was among the most highly down-regulated ncRNAs with a Log_2_FC of -2.66.

**Table 3:** Differentially expressed ncRNAs upon exposure to lethal dose of H_2_O_2_.

NC RNA	chromosome	Log_2_Fc	Adjusted p-value	Start-End	Overlapping ORF^a^	Upstream ORF^a^	Downstream ORF^a^	*perR* mutant^b^
***Up-regulated***								
rh34	NZ_CP011933.1	1.218	1.23e-30	18632–19081	LIMLP_19345	LEPIMA _p0012	LIMLP_19350	
rh36	NZ_CP011933.1	1.368	4.02e-37	20723–21129	NA	LIMLP_19355	LIMLP_19360	
rh47	NZ_CP011931.1	1.150	2.80e-57	37221–37455	NA	LIMLP_00160	LIMLP_00165	
rh49	NZ_CP011932.1	1.013	2.50e-47	39710–39825	LEPIMA_CII0041	LIMLP_18000	LIMLP_18005	
rh57^§^	NZ_CP011933.1	1.629	1.95e-69	23941–24050	LEPIMA _p0025	LIMLP_19380	LIMLP_19385	
rh82	NZ_CP011933.1	1.362	3.50e-37	32192–32351	NA	LIMLP_19435	LEPIMA _p0038	
rh97	NZ_CP011932.1	1.170	3.03e-57	66008–66133	LIMLP_18130	LIMLP_18125	LEPIMA_CII0073	
rh178	NZ_CP011933.1	1.195	2.92e-22	57959–58185	NA	LEPIMA _p0081	LIMLP_19600	
rh179	NZ_CP011933.1	1.082	7.04e-14	58957–59117	LEPIMA _p0083	LIMLP_19600	LIMLP_19605*	
rh183	NZ_CP011931.1	1.557	7.61e-80	128941–129006	NA	LIMLP_00580	LIMLP_00585	
rh184	NZ_CP011931.1	1.228	2.09e-66	129119–129450	LIMLP_00585	LIMLP_00580	LIMLP_00595	
rh199	NZ_CP011933.1	1.009	7.80e-24	63659–64935	LEPIMA_p0089, LIMLP_19625, LIMLP_19630	LIMLP_19620	LIMLP_19635	
rh210	NZ_CP011931.1	1.231	1.22e-61	158886–159090	NA	LIMLP_00700*	LIMLP_00705	
rh219	NZ_CP011932.1	1.337	2.22e-97	147192–147336	LIMLP_18455**	LIMLP_18450	LIMLP_18460**	
rh288	NZ_CP011931.1	3.812	0.00	197282–197352	LIMLP_00895*	LIMLP_00890	LEPIMA_CI0185	Up-regulated
rh349	NZ_CP011932.1	1.638	1.98e-120	256577–256639	NA	LEPIMA_CII0243	LIMLP_18855	
rh402	NZ_CP011932.1	1.517	1.31e-92	291497–291605	LIMLP_18995	LIMLP_18990**	LIMLP_19000*	
rh449	NZ_CP011931.1	2.512	0.00	351592–351649	NA	LIMLP_01545*	LIMLP_01550*	
rh488	NZ_CP011932.1	1.184	1.11e-48	352594–352673	LIMLP_19275	LIMLP_19270	LIMLP_19280	
rh490	NZ_CP011932.1	1.277	2.18e-49	353695–353790	LIMLP_19280	LIMLP_19275	LIMLP_19285	
rh593	NZ_CP011931.1	1.362	1.44e-43	471763–471828	LIMLP_02010**	LIMLP_02005	LEPIMA_CI0422	
rh608	NZ_CP011931.1	2.476	0.00	479394–479448	LIMLP_02045*	LIMLP_02040*	LIMLP_02050	
rh625	NZ_CP011931.1	1.077	9.15e-49	499440–499814	LIMLP_02100	LIMLP_02095	LIMLP_02105*	
rh637^#^	NZ_CP011931.1	1.663	2.16e-137	501388–501477	NA	LIMLP_02105*	LIMLP_02110	
rh786	NZ_CP011931.1	1.135	6.17e-50	632024–632186	LIMLP_02580	LIMLP_02575	LIMLP_02585	
rh859^#^	NZ_CP011931.1	4.248	0.00	683752–684074	NA	LIMLP_02795*	LEPIMA_CI0612	Up-regulated
rh1048	NZ_CP011931.1	1.059	3.63e-43	846807–846960	LIMLP_03520	LIMLP_03515	LIMLP_03525	
rh1167	NZ_CP011931.1	1.070	2.97e-37	943926–943989	LIMLP_03935	LIMLP_03930	LIMLP_03940	
rh1192	NZ_CP011931.1	2.197	5.78e-214	975150–975213	LIMLP_04030	LIMLP_04025	LIMLP_04035	
rh1210 (RF00059)	NZ_CP011931.1	1.236	3.67e-37	995004–995065	NA	LIMLP_04090	LIMLP_04095**	
rh1269	NZ_CP011931.1	2.164	2.97e-202	1038822–1038876	LIMLP_04290	LIMLP_04285	LIMLP_04295*	
rh1270	NZ_CP011931.1	1.491	2.13e-108	1039034–1039628	LIMLP_04295*, LEPIMA_CI0938	LIMLP_04290	LIMLP_04300	
rh1429	NZ_CP011931.1	1.720	3.15e-90	1181397–1181456	LIMLP_04840	LIMLP_04830	LIMLP_04845	
rh1498	NZ_CP011931.1	1.288	1.08e-46	1260173–1260234	NA	LEPIMA_CI1128	LIMLP_05125*	
rh1641	NZ_CP011931.1	2.013	2.50e-145	1386755–1386830	LIMLP_05625	LIMLP_05620*	LIMLP_05630	
rh1807	NZ_CP011931.1	2.928	0.00	1531048–1531289	LIMLP_06235	LIMLP_06230	LIMLP_06240	
rh2088	NZ_CP011931.1	2.000	1.66e-149	1780300–1780403	LIMLP_07195	LEPIMA_CI1612	LIMLP_07200	
rh2227	NZ_CP011931.1	3.130	0.00	1892070–1892135	NA	LIMLP_07695	LIMLP_07700	
rh2395	NZ_CP011931.1	1.877	2.09e-123	2013277–2013341	LIMLP_08295	LIMLP_08290	LIMLP_08300	
rh2487	NZ_CP011931.1	1.230	5.68e-66	2083779–2083898	LIMLP_08585	LEPIMA_CI1903	LIMLP_08590*	Down-regulated
rh2961	NZ_CP011931.1	1.974	8.15e-150	2474618–2474668	LIMLP_10350	LIMLP_10345	LIMLP_10355	
rh3130^#^	NZ_CP011931.1	7.189	0.00	2612368–2612495	LEPIMA_CI2416	LIMLP_10975*	LEPIMA_CI2417	
rh3147	NZ_CP011931.1	1.230	8.22e-39	2625684–2625735	LIMLP_11030	LIMLP_11025	LEPIMA_CI2429	
rh3352^#^	NZ_CP011931.1	7.653	0.00	2787780–2787953	LIMLP_11710*	LIMLP_11705	LIMLP_11715*	
rh3535	NZ_CP011931.1	1.016	1.77e-49	2958335–2958610	NA	LIMLP_12420	LIMLP_12425*	
rh3538	NZ_CP011931.1	1.000	2.78e-21	2958938–2959002	LIMLP_12425*	LIMLP_12420	LIMLP_12430*	
rh3726	NZ_CP011931.1	1.010	5.62e-46	3123653–3123980	LIMLP_13150	LIMLP_13145*	LIMLP_13155	
rh3831	NZ_CP011931.1	1.060	1.74e-26	3214862–3214919	LIMLP_13525	LIMLP_13520	LIMLP_13530	
rh3871	NZ_CP011931.1	2.133	2.34e-261	3253035–3253139	LIMLP_13675	LIMLP_13670*	LIMLP_13680	
rh3894	NZ_CP011931.1	3.784	0.00	3271638–3271704	NA	LIMLP_13765*	LIMLP_13770	
rh4111	NZ_CP011931.1	1.340	2.82e-76	3446561–3446742	LIMLP_14535	LIMLP_14530	LEPIMA_CI3186	
rh4124	NZ_CP011931.1	1.233	3.41e-66	3459090–3459232	NA	LIMLP_14580	LIMLP_14585*	
rh4168	NZ_CP011931.1	1.244	4.53e-58	3487314–3487435	NA	LIMLP_14710	LIMLP_14715*	
rh4281	NZ_CP011931.1	1.627	1.38e-71	3584015–3584072	LIMLP_15080**	LIMLP_15075**	LIMLP_15085	
rh4345	NZ_CP011931.1	1.765	8.56e-126	3664279–3664343	LIMLP_15310	LIMLP_15305	LIMLP_15315**	
rh4413^#^	NZ_CP011931.1	3.507	0.00	3721204–3721564	NA	LIMLP_15540*	LIMLP_15545	
rh4459^#^	NZ_CP011931.1	1.059	6.95e-29	3755947–3756010	NA	LIMLP_15710	LEPIMA_CI3455	
rh4542	NZ_CP011931.1	2.748	0.00	3822746–3823025	LIMLP_16010	LIMLP_16005	LIMLP_16015*	
rh4545	NZ_CP011931.1	1.979	1.54e-233	3825144–3825319	NA	LIMLP_16015*	LIMLP_16025*	
rh4746	NZ_CP011931.1	1.222	1.77e-36	3987147–3987296	LEPIMA_CI3684	LIMLP_16760	LIMLP_16765*	
rh4747	NZ_CP011931.1	1.178	9.32e-60	3987366–3987576	LEPIMA_CI3684	LIMLP_16760	LIMLP_16765*	
rh4854 (RF00174)	NZ_CP011931.1	1.023	9.20e-41	4078407–4078514	NA	LIMLP_17135	LIMLP_17140	
rh5034	NZ_CP011931.1	1.628	2.24e-72	4229144–4229208	NA	LIMLP_17780	LIMLP_17785	
***Down-regulated***								
rh38	NZ_CP011932.1	-1.078	2.65e-08	32083–32148	LIMLP_17965	LIMLP_17960	LIMLP_17970	
rh81	NZ_CP011931.1	-1.246	3.21e-11	67349–67433	NA	LIMLP_00285	LIMLP_00290	
rh278	NZ_CP011932.1	-1.188	8.73e-13	202039–202120	NA	LIMLP_18675	LEPIMA_CII0202	
rh331	NZ_CP011931.1	-1.137	9.15e-08	245678–245742	NA	LIMLP_01140	LEPIMA_CI0236	
rh411	NZ_CP011931.1	-1.854	1.53e-65	310470–310529	NA	LIMLP_01410**	LIMLP_01415**	
rh418	NZ_CP011931.1	-1.395	9.73e-15	317250–317316	NA	LIMLP_01445	LIMLP_01450	
rh429	NZ_CP011932.1	-1.375	2.93e-11	311436–311731	NA	LIMLP_19090	LIMLP_19095	
rh589	NZ_CP011931.1	-1.000	3.55e-07	469227–469400	NA	LIMLP_01995	LIMLP_02000	
rh685	NZ_CP011931.1	-1.613	1.73e-40	541558–541624	NA	LEPIMA_CI0489	LIMLP_02275	
rh697	NZ_CP011931.1	-1.276	3.61e-11	549563–549676	NA	LIMLP_02295	LIMLP_02300	
rh698	NZ_CP011931.1	-1.170	8.45e-09	549730–549828	NA	LIMLP_02295	LIMLP_02300	
rh711	NZ_CP011931.1	-1.080	1.30e-07	562570–562701	NA	LIMLP_02335	LIMLP_02340	
rh736	NZ_CP011931.1	-1.474	3.95e-17	582458–582528	NA	LIMLP_02395**	LIMLP_02400**	
rh753	NZ_CP011931.1	-1.226	1.94e-13	602773–602842	NA	LIMLP_02460	LIMLP_02465	
rh784	NZ_CP011931.1	-1.103	1.10e-10	630185–630381	LIMLP_02570	LIMLP_02565	LIMLP_02575	
rh967	NZ_CP011931.1	-2.662	8.54e-202	786700–786893	NA	LIMLP_03220**	LIMLP_03225	
rh1008	NZ_CP011931.1	-1.145	5.44e-10	819424–819662	NA	LIMLP_03375	LIMLP_03380	
rh1101	NZ_CP011931.1	-2.684	4.82e-295	888430–888480	NA	LIMLP_03700	LIMLP_03705**	
rh1102	NZ_CP011931.1	-2.149	2.11e-80	888546–888608	NA	LIMLP_03700	LIMLP_03705**	
rh1140	NZ_CP011931.1	-1.188	5.61e-08	920214–921272	NA	LIMLP_03840	LIMLP_03845	
rh1142	NZ_CP011931.1	-1.157	6.58e-10	920631–920965	NA	LIMLP_03840	LIMLP_03845	
rh1253	NZ_CP011931.1	-1.608	1.10e-30	1025093–1025156	LEPIMA_CI0924	LEPIMA_CI0923	LEPIMA_CI0925	
rh1282	NZ_CP011931.1	-1.367	4.09e-15	1046519–1046574	LEPIMA_CI0946	LIMLP_04325**	LIMLP_04330	
rh1299	NZ_CP011931.1	-1.054	9.24e-08	1057522–1057700	LEPIMA_CI0958	LIMLP_04385	LIMLP_04390	
rh1382	NZ_CP011931.1	-1.036	1.69e-05	1129722–1129784	NA	LEPIMA_CI1022	LEPIMA_CI1023	
rh1651	NZ_CP011931.1	-1.354	4.48e-12	1392744–1392884	LIMLP_05660	LIMLP_05655	LIMLP_05665	
rh1880	NZ_CP011931.1	-1.896	6.86e-62	1592557–1592621	LEPIMA_CI1441	LIMLP_06480**	LEPIMA_CI1442	
rh2038	NZ_CP011931.1	-1.252	1.81e-14	1734004–1734144	NA	LIMLP_07030	LIMLP_07035	
rh2114	NZ_CP011931.1	-1.206	2.39e-12	1799567–1799634	NA	LIMLP_07290	LIMLP_07295	
rh2170	NZ_CP011931.1	-1.047	1.58e-06	1854827–1854890	LEPIMA_CI1676	LIMLP_07495	LIMLP_07500	
rh2222	NZ_CP011931.1	-1.199	1.09e-12	1896532–1896782	NA	LIMLP_07715	LIMLP_07725	
rh2311	NZ_CP011931.1	-1.115	1.01e-06	1954525–1954640	LIMLP_07975	LIMLP_07970**	LIMLP_07980	
rh2578	NZ_CP011931.1	-1.730	2.54e-44	2165614–2165832	LIMLP_08925	LIMLP_08920	LIMLP_08930**	
rh2850	NZ_CP011931.1	-1.378	1.21e-14	2378010–2378075	LEPIMA_CI2192	LIMLP_09945	LIMLP_09950	
rh2882	NZ_CP011931.1	-1.299	2.18e-12	2398625–2398696	NA	LIMLP_10050**	LEPIMA_CI2213	
rh3186	NZ_CP011931.1	-1.963	5.74e-64	2658407–2658646	NA	LIMLP_11175**	LIMLP_11180**	
rh3190	NZ_CP011931.1	-1.874	6.42e-47	2656130–2656312	NA	LIMLP_11170**	LIMLP_11175**	
rh3335	NZ_CP011931.1	-1.382	4.99e-21	2764692–2764792	NA	LIMLP_11630	LIMLP_11635	
rh3711	NZ_CP011931.1	-2.030	5.35e-87	3116206–3116269	NA	LIMLP_13120	LEPIMA_CI2881	
rh3945	NZ_CP011931.1	-1.465	7.13e-22	3308829–3309056	LEPIMA_CI3062	LIMLP_13935	LIMLP_13940	
rh3946	NZ_CP011931.1	-1.126	3.52e-10	3309111–3309181	NA	LEPIMA_CI3062	LIMLP_13940	
rh4140	NZ_CP011931.1	-1.438	7.92e-17	3467363–3467432	NA	LIMLP_14615**	LIMLP_14620**	
rh4178	NZ_CP011931.1	-1.509	2.89e-18	3496010–3496183	LEPIMA_CI3239	LIMLP_14745**	LIMLP_14750	
rh4218	NZ_CP011931.1	-1.174	6.15e-08	3532306–3532358	NA	LEPIMA_CI3268	LIMLP_14880	
rh4253	NZ_CP011931.1	-1.332	9.51e-14	3554072–3554567	NA	LIMLP_14970**	LIMLP_14975	
rh4254	NZ_CP011931.1	-1.246	1.77e-09	3554619–3554683	NA	LIMLP_14970**	LIMLP_14975	
rh4493	NZ_CP011931.1	-1.115	2.32e-09	3783194–3783362	NA	LIMLP_15840	LIMLP_15845	
rh4549	NZ_CP011931.1	-1.821	7.63e-51	3827129–3827377	LEPIMA_CI3525	LIMLP_16030	LIMLP_16035**	
rh4607	NZ_CP011931.1	-1.280	4.56e-14	3879350–3879511	LIMLP_16285	LIMLP_16280	LIMLP_16290	
rh4763	NZ_CP011931.1	-1.116	3.50e-03	3984140–3984338	LIMLP_16745**	LIMLP_16740	LIMLP_16750	
rh4894	NZ_CP011931.1	-1.137	3.70e-11	4116602–4116674	LIMLP_17290	LIMLP_17285	LIMLP_17295	
rh4918	NZ_CP011931.1	-1.256	5.77e-17	4133590–4133654	NA	LIMLP_17350	LIMLP_17355	
rh4938	NZ_CP011931.1	-1.064	3.10e-08	4155394–4155481	LIMLP_17425**	LIMLP_17420**	LIMLP_17430**	

Significantly differentially-expressed ncRNAs upon 1h exposure to 1 mM H_2_O_2_ with Log_2_FC cutoff of ± 1.0 and a p-value cutoff of 0.05.

^a^ Gene numeration is according to to Satou et al. [17].

^b^ Differential expression of ncRNA upon inactivation of *perR* (M776 mutant) (see S5 Table).

^#^ ncRNAs significantly up-regulated upon a 30 min exposure to 10 μM H_2_O_2_ (Log_2_FC cutoff of 1, p-value cutoff of 0.05, see S5 Table).

^§^ ncRNA significantly down-regulated upon a 30 min exposure to 10 μM H_2_O_2_ (Log_2_FC cutoff of -1, p-value cutoff of 0.05, see S5 Table).

* ORFs significantly up-regulated by RNASeq analysis (Log_2_FC cutoff of 1, adjusted p-value cutoff of 0.05).

** ORFs significantly down-regulated by RNASeq analysis (Log_2_FC cutoff of -1, adjusted p-value cutoff of 0.05).

NA, non-applicable

The Rfam classification of ncRNAs is indicated into parenthesis.

Several of the ncRNAS whose expression was up- or down-regulated in the presence of hydrogen peroxide were located in the vicinity or overlapped ORFs that were also up- or down-regulated in the same conditions. For instance, the rh3130 and rh859, among the most highly up-regulated ncRNAs, were in the vicinity of Hsp20 and CCP-encoding ORFs (LIMLP_10970–10975 and LIMLP_02795, respectively), three genes whose expression was greatly increased in the presence of hydrogen peroxide (Tables 1 and 3 and Fig 3). LIMLP_05620, LIMLP_13670, and LIMLP_13765 were three up-regulated ORFs upon exposure to hydrogen peroxide that have a downstream ncRNA (rh1641, rh3871, and rh3894, respectively). The up-regulated rh288 overlapped with the H_2_O_2_-induced LIMLP_00895, an ORF located in the prophage locus 1 (Table 3). This tendency was also observed with down-regulated ncRNAs. Rh411, rh967, rh1101, rh1102, rh1880, rh3186, and rh4281 ncRNAs were also located downstream or upstream, or overlapped ORFs whose expression was decreased in the presence of hydrogen peroxide (Table 3).

Three ncRNAs were noticeably differentially expressed upon *perR* inactivation. Rh288 and rh859 were up-regulated and rh1263 (located in the intergenic region upstream the TonB/ExbD_2_/ExbB-encoding operon, LIMLP_04255–04240) was significantly down-regulated in the *perR* mutant (S5 Table). Interestingly, the ncRNA rh859 was still up-regulated in the *perR* mutant upon exposure to H_2_O_2_ (S5 Table). This indicates that the rh859 up-regulation induced by the exposure of WT *Leptospira* to H_2_O_2_ occurs to some extent independently of the presence of PerR.

Most of the predicted ncRNAs show little homology with well-characterized RNAs families of the RFam database (S6 Table). However, this study has allowed the identification of a putative TPP riboswitch (rh1210; RFam 00059), three putative cobalamin riboswitches (rh1913, rh3382, rh4854; RFam 00174), a putative AsrC (Antisense RNA of rseC) (rh2876; RFam 02746) and a putative ligA thermometer (rh1488; RFam02815). Only the putative TPP (rh1210) and cobalamin (rh4854) riboswitches were up-regulated upon *Leptospira* exposure to H_2_O_2_. Further experiments will be needed to confirm the existence of these putative ncRNAs and establish their function in *Leptospira* physiology and virulence. Genetic manipulation of pathogenic *Leptospira* is still a challenge and functional studies in these bacteria mainly relies on random transposon insertion. Our laboratory has constructed a transposon mutant library [31], however no mutant in the putative ncRNAs is yet available in our random transposon mutant library.

Altogether, these findings indicate that exposure of *Leptospira* to 1 mM H_2_O_2_ triggers a drastic change in the expression of putative ncRNAs that correlates with dramatic changes in coding sequence expression.

### Role of the PerR-regulated genes in defenses against ROS and virulence in *Leptospira*

RNA-Seq experiments have allowed the identification of differentially expressed ORFs in the presence of peroxide and upon *perR* inactivation. These ORFs might encode factors required for the adaptation of pathogenic *Leptospira* to ROS and an important question is to experimentally establish and understand the role of these factors in this adaptation. Several mutants inactivated in differentially-expressed ORFs upon exposure to H_2_O_2_ or upon *perR* inactivation were available in our transposon mutant library.

**Fig 6:**
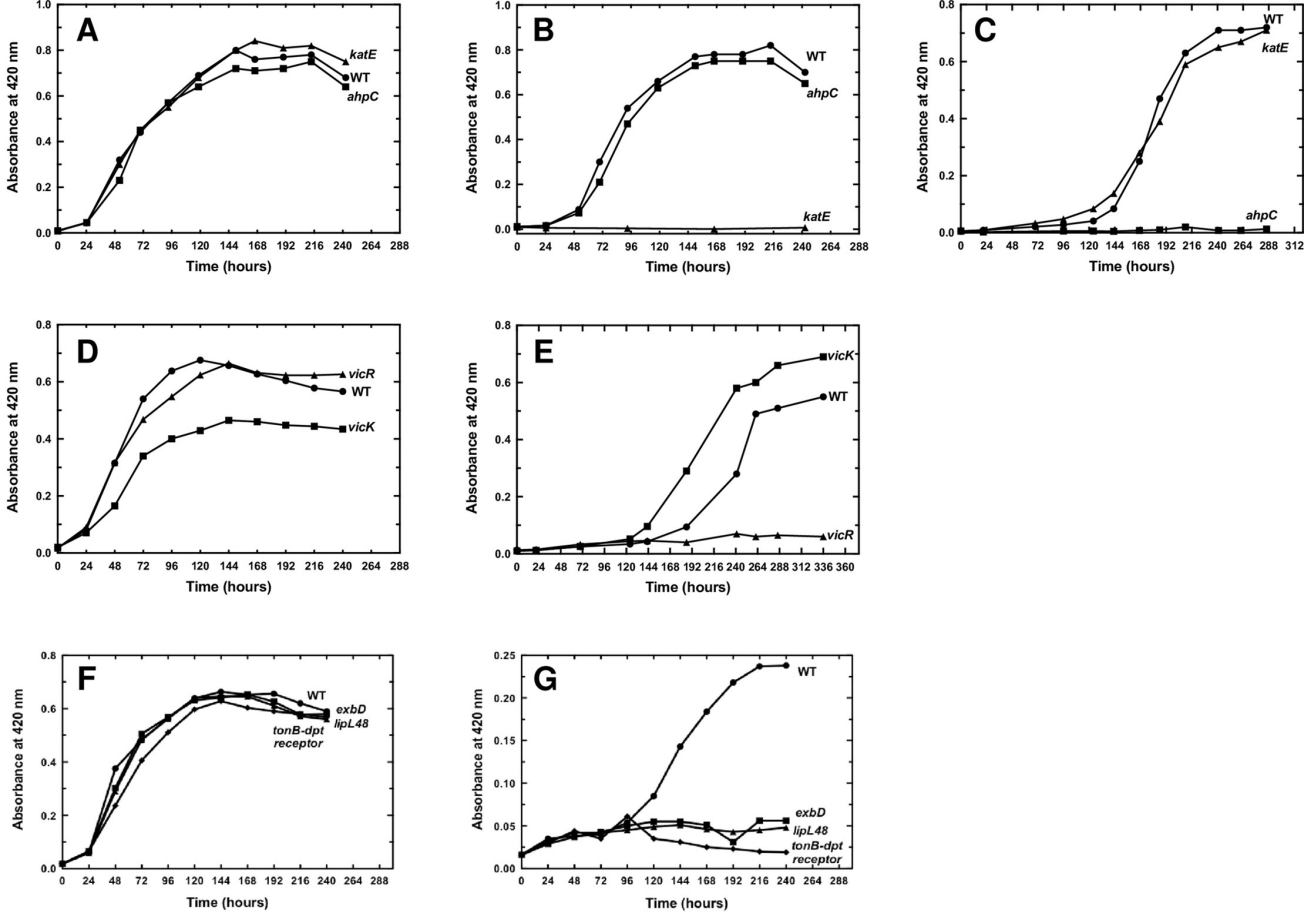
Effect of the inactivation of PerR-controlled genes on *Leptospira* growth in the presence of ROS. *L*. *interrogans* WT, *katE* (Man69) and *ahpC* (Man1368), *vicK* (Man1448) and *vicR* (Man899), *tonB-dpt receptor* (Man1022), *exbD* (Man782), and *lipl48* (Man1089) mutant strains were cultivated in EMJH medium (A, D, F) or in the presence of 2 mM H_2_O_2_ (B) or of 2 μM paraquat (C, E, G). Growth was assessed by measure of absorbance at 420 nm.

Catalase, AhpC, and CCP were the peroxidases up-regulated in the presence of H_2_O_2_ and repressed by PerR. Only *katE* and *ahpC* mutants were available in the transposon mutant library and we have studied the ability of these mutants to grow in the presence of H_2_O_2_ and paraquat, a superoxide-generating compound. These two mutants had a comparable growth rate in EMJH medium (Fig 6A) but when the medium was complemented with 0.5 mM H_2_O_2_, the ability of the *katE* mutant to divide was dramatically impaired (Fig 6B). The growth rate of the *ahpC* mutant in the presence of H_2_O_2_ was comparable to that of the WT strain (Fig 6B). When the EMJH medium was complemented with 2 μM paraquat, the growth of the *ahpC* mutant was considerably reduced, indicating a high sensitivity to superoxide (Fig 6C).

**Fig 7:**
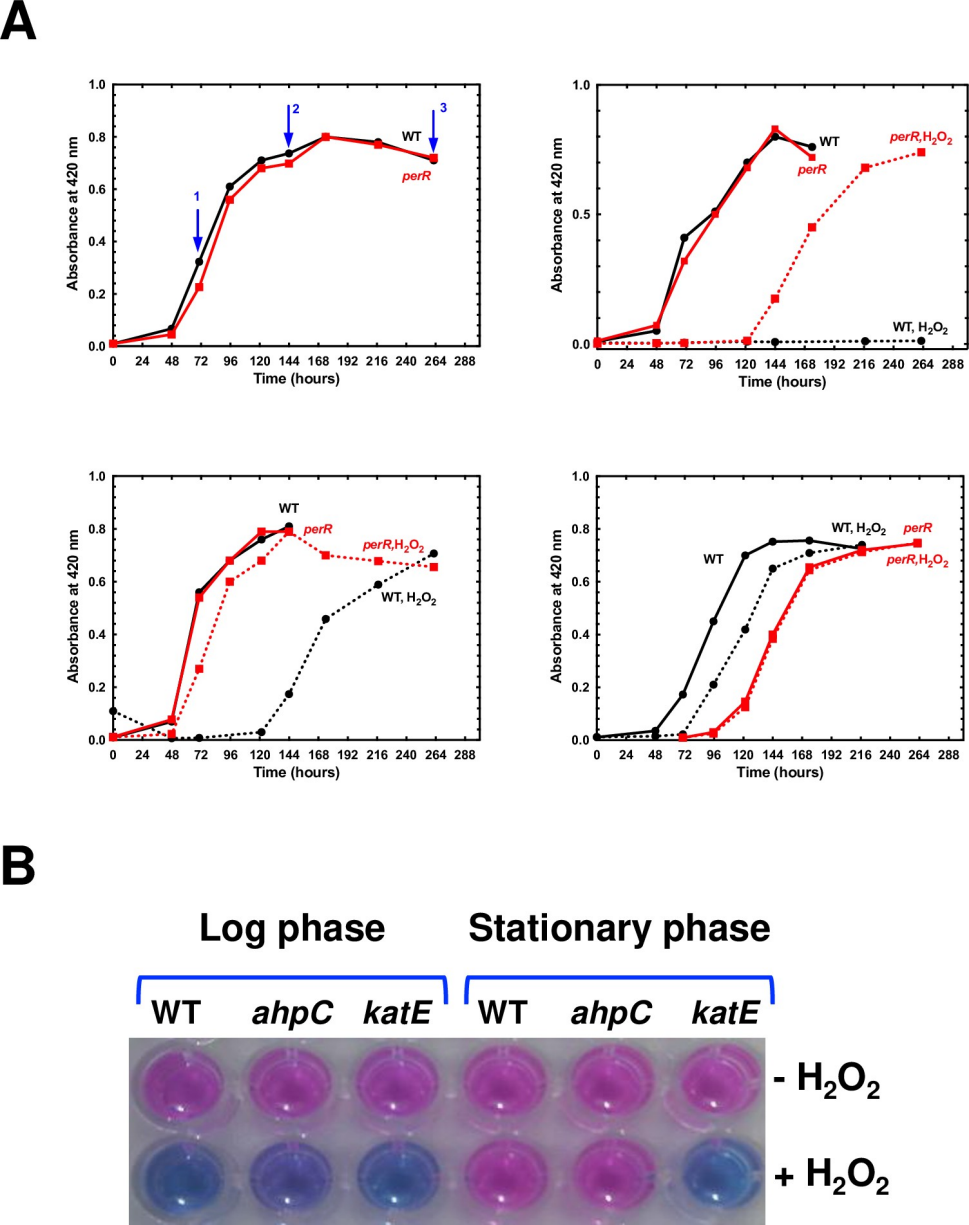
Role of catalase and AhpC in the stationary phase-adapted *Leptospira* tolerance to hydrogen peroxide. (A) *L*. *interrogans* WT (black line) and *perR* mutant (M776) (red line) strains were cultivated in EMJH medium and samples were taken at the exponential phase (at OD_420_ nm ≈ 0.3, left upper panel, blue arrow 1), at the entry of stationary phase (at OD_420_ nm ≈ 0.7, left upper panel, blue arrow 2), and at late stationary phase (at OD_420_ nm ≈ 0.7, 5 days after the entry in stationary phase, left upper panel, blue arrow 3) and used to inoculate a new EMJH medium in the absence (plain line) or presence of 2 mM H_2_O_2_ (dashed line). The growth curve with samples taken in the exponential phase (samples 1), in the entry of stationary phase (samples 2) and at late stationary phase (samples 3) are represented in the right upper, the left lower, and the right lower panels, respectively. (B) *L*. *interrogans* WT, *katE* (Man69) and *ahpC* (Man1368) mutant strains were cultivated in EMJH medium until the exponential or stationary phases and incubated for 30 min in the absence or presence of 10 mM H_2_O_2_. Cell viability was assessed by the ability of the cells to reduce the blue rezasurin into a pink resorufin using the Alamar Blue assay as described in the Material and Methods section.

In other bacteria including *E*. *coli* and *B*. *subtilis*, *katE* is produced in higher amount during stationary phase [32,33], and in order to further characterize the role of *katE* in *Leptospira* survival under oxidative stress, we investigated the survival of stationary phase-adapted *Leptospira* in the presence of H_2_O_2_. *L*. *interrogans* WT cells were cultivated in EMJH medium and samples were harvested in the logarithmic phase (Fig 7A, sample 1), at the entry in stationary phase (Fig 7A, sample 2) and in late stationary phase (Fig 7A, sample 3). Each sample was used to inoculate a new batch of EMJH medium in the absence or presence of 2 mM H_2_O_2_. As seen in Fig 7A, when EMJH was inoculated with *Leptospira* WT strain at logarithmic phase, *Leptospira* were not able to divide in the presence of 2 mM H_2_O_2_. However, when the culture medium was inoculated with *Leptospira* WT strain at the beginning of the stationary phase, *Leptospira* acquired a greater resistance to 2 mM H_2_O_2_ as seen by their ability to grow (Fig 7A). An even higher ability to grow in the presence of a deadly dose of H_2_O_2_ was observed when the EMJH medium was inoculated with *Leptospira* at late stationary phase (Fig 7A). This indicates that *Leptospira* acquire a higher tolerance to hydrogen peroxide at stationary phase. Interestingly, this acquired tolerance to H_2_O_2_ was independent of PerR since the *perR* mutant also acquired a higher ability to grow in the presence of 2 mM H_2_O_2_ when at stationary phase (Fig 7A). In order to determine which peroxidase was responsible for this acquired tolerance to H_2_O_2_, the survival of WT, *ahpC* and *katE* mutant strains was tested in logarithmic phase and was compared with that in stationary phase. As seen in Fig 7B, a 30 min exposure to 10 mM H_2_O_2_ led to dramatic loss of survival of all strains at logarithmic phase. WT and *ahpC* mutant strains were able to acquire a higher resistance to H_2_O_2_ when placed at stationary phase whereas the *katE* mutant did not. Therefore, *katE* is essential for the stationary phase-acquired resistance to H_2_O_2_ and this probably involves another regulation mechanism than that exerted by PerR.

Among the genes repressed by PerR, only mutants inactivated in LIMLP_04245 (*exbD*), LIMLP_04270 (*tonB-dpt receptor*), LIMLP_04280 (*lipl48*), LIMLP_16720 (*vicR*), and LIMLP_16725 (*vicK*), were available in the transposon mutant library. All these mutants but *vicK* had a growth rate comparable to that of the WT strain in EMJH medium (Fig 6D and 6F). Despite the fact that *vicK* had a reduced ability to divide in EMJH medium, this mutant strain had a slightly greater resistance to 2 μM paraquat than that of the WT (Fig 6E). In the same condition, the *vicR*, *exbD*, *tonB-dpt receptor*, and *lipl48* mutant strains had a lower ability to grow than the WT strain (Fig 6E and 6G). Altogether, these findings suggest that some of the PerR-repressed ORFs are involved in *Leptospira* defense against superoxide.

**Fig 8:**
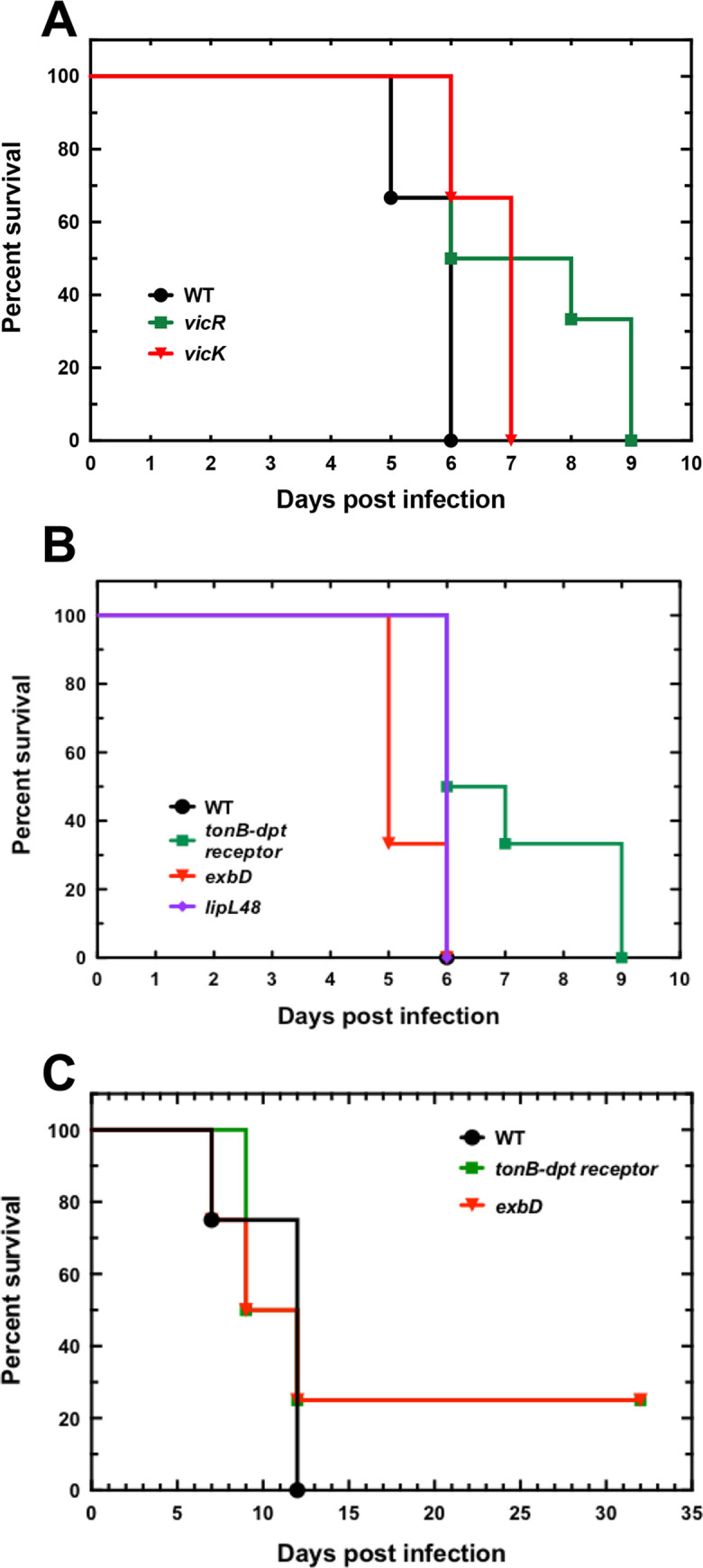
Role of PerR-controlled ORFs in *Leptospira* virulence. 10^6^ of WT, *vicK* (Man1448) and *vicR* (Man899) mutant strains (A), or the *tonB-dpt receptor* (Man1022), *exbD* (Man782), *lipl48* (Man1089) mutant strains (B) or 10^4^ of WT, *exbD* (Man782), or the *tonB-dpt receptor* (Man1022) mutant strains (C) were injected intraperitoneally in hamsters (n = 4–8) as described in Material and Methods section.

Catalase has been shown to be essential for *Leptospira* virulence [9]. We investigated whether other PerR-controlled genes were also required for *Leptospira* virulence. The different mutants were used in infection experiments in the acute model for leptospirosis. *VicK*, *exbD*, and *lipl48* mutants did not exhibit dramatically altered virulence when 10^6^ bacteria were injected peritoneally in hamsters (Fig 8A and 8B). In order to further challenge the role of the TonB-dependent transport system in *Leptospira* virulence, we tested whether a lower dose of infection with the *tonB-dpt receptor* and *exbD* mutants would result in a virulence attenuation. As seen in Fig 8C, when 10^4^ bacteria were injected peritoneally in hamsters, animals infected with the *exbD* mutant exhibited 25% survival at 32 days post infection with no sign of leptospirosis. However, this slight virulence attenuation is not statistically significant. Therefore, inactivation of the TonB-dependent transport system or of the two-component system VicKR does not have a drastic consequence on *Leptospira* virulence in the acute model of infection in the conditions used in this study. These mechanisms do not have a pivotal role in *Leptospira* during infection or redundant activities compensate for their absence. Experiments using other infection routes (ocular or subcutaneous routes) might result in different outcomes.

## Discussion

Reactive oxidative species are powerful and efficient weapons used by the host innate immunity response to eliminate pathogens. The ability of pathogenic *Leptospira* to detoxify hydrogen peroxide, one of the ROS produced upon *Leptospira* infection and pathogenicity, is essential for these pathogenic bacteria virulence [9]. Because *Leptospira* are also environmental aerobic bacteria, they will also face low concentrations of ROS endogenously produced through the respiratory chain or present in the outside environment. The present study has used RNA-Seq technology to determine the response of pathogenic *Leptospira* to hydrogen peroxide. Our study allowed, for the first time, a genome-wide identification of differentially-expressed factors in response to exposure of pathogenic *Leptospira* to H_2_O_2_.

**Fig 9:**
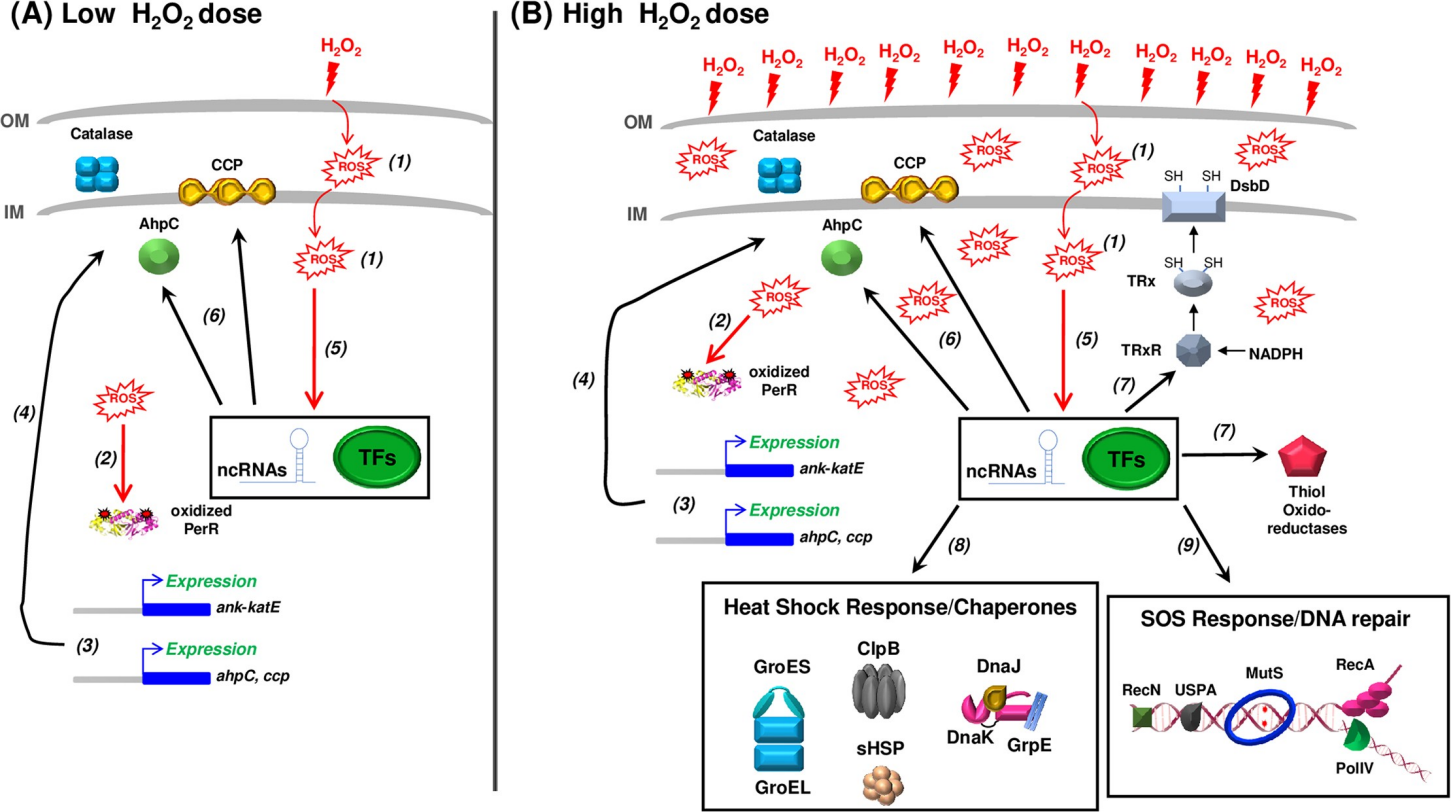
Schematic representation of the pathogenic *Leptospira* response to hydrogen peroxide. In the presence of low H_2_O_2_ dose (A), ROS are produced in the cell *(1)*, PerR is oxidized *(2)* and dissociates from DNA regions in the locus of the three peroxidase-encoding genes (*ank-katE* operon, *ahpC*, *ccp*), leading to their derepression *(3)* and increased production of catalase, AhpC and CCP *(4)*. Other transcriptional regulator (TFs) and non-coding RNAs whose expression is affected in the presence of ROS *(5)* probably participate in the H_2_O_2_-induced increase of AhpC and CCP production *(6)*. As a result, the activities of catalase, AhpC and CCP allow maintaining ROS at a harmless level. The increased expression of heme biosynthesis genes, which is PerR-independent and probably participates in the peroxidase activities of catalase, AhpC and CCP, is not represented here. When the level of ROS overwhelms the detoxification capacity of the up-regulated peroxidases and becomes damaging for the cellular constituents (B), in addition of a higher production of catalase, AhpC and CCP, other machineries are up-regulated such as thiol oxido-reductases (including thioredoxin, DsbD, etc.) *(7)*, molecular chaperones *(8)* and DNA repair proteins *(9)*. The increased expression of the aforementioned machineries is PerR-independent and probably involves other transcriptional regulators (TFs) and noncoding RNAs.

*L*. *interrogans* were exposed to sublethal (10 μM) and lethal (1 mM) doses of hydrogen peroxide that could mimic the hydrogen peroxide concentrations encountered inside a host. Our findings indicate that the peroxide stress response is temporal and dose-dependent. *L*. *interrogans* can sense and rapidly respond to H_2_O_2_ concentrations as low as 10 μM by up-regulating the catalase and two peroxidases, AhpC and CCP (Fig 9A). Heme biosynthesis-encoding genes were also up-regulated probably because catalase and CCP have heme-dependent peroxidase activities. These three peroxidases are the first-line of defense allowing detoxification of H_2_O_2_, and among these three enzymes, catalase has a major role in protecting *L*. *interrogans* from the deadly effect of hydrogen peroxide, during logarithmic phase but also during stationary phase. Arias *et al*. [22] showed that *E*. *coli* cells overexpressing the *L*. *interrogans* AhpC displayed a higher survival in the presence of H_2_O_2_ and *tert*-Butyl hydroperoxide. In our study, an *ahpC* mutant did not exhibit an altered tolerance toward H_2_O_2_; instead, this mutant had a lower ability to grow in the presence of paraquat, a superoxide-generating chemical. Although we cannot rule out that the inactivation of *ahpC* triggers an increase in catalase activity to compensate the absence of AhpC, our findings might indicate a role of this peroxidase in detoxification of superoxide or of H_2_O_2_ produced from the catabolism of superoxide. The role of CCP in degrading H_2_O_2_ in pathogenic *Leptospira* has never been investigated. Whether CCP fulfills such a role or whether CCP rather acts as an electron acceptor for the respiratory chain, as demonstrated in *E*. *coli* [34], will require obtaining a deletion mutant by allelic exchange since a *ccp* mutant was not available in the transposon mutant library.

The up-regulation of catalase, AhpC and CCP is probably sufficient to rapidly degrade H_2_O_2_ and avoid accumulation of ROS inside the cells. However, when the H_2_O_2_ level is high, as occurs when *L*. *interrogans* are exposed to 1 mM H_2_O_2_ (Fig 9B), it could overwhelm the H_2_O_2_ detoxification machinery. Additional enzymes with a putative role as antioxidants and/or in repair of oxidized cysteines in proteins are also up-regulated (including thioredoxin, glutaredoxin, DsbD and Bcp-like proteins). The induction of several genes of the LexA regulon (*recA*, *recN*, *dinP*) and other genes with putative role in DNA repair (*mutS*, *radC*) suggests that these concentrations of H_2_O_2_ induced oxidative damage to DNA and a need for the SOS response. Also, canonical molecular chaperones (DnaK/J/GrpE, GroEL/ES, ClpB, small Hsps) were dramatically more expressed, suggesting that 1 mM H_2_O_2_ results in protein aggregation and unfolding.

Several of the factors whose expression is up-regulated upon exposure to H_2_O_2_ were also up-regulated when *Leptospira* are cultivated in DMC implemented in rat peritoneal cavities [16]. Among those were the peroxidases AhpC, CCP, their repressor PerR, RCC1, as well as genes encoding DNA repair proteins (LIMLP_11400, LIMLP_16520, and LIMLP_16525). This suggests that the conditions used in the present study properly reproduce the oxidative stress encountered by *Leptospira* when infecting a mammalian host. Many H_2_O_2_-induced ORFs identified in our study have been shown to be also up-regulated upon other host-related conditions such as at the host temperature of 37°C (Catalase, GroEL/ES, DnaK/J/GrpE, small HSPs, ClpB, RadC) [35–37], host osmolarity (RadC, DsbD, LIMLP_00770, and LIMLP_16520) [38], or under iron-limited condition (LIMLP_08410, LIMLP_14160, Imelysin (LIMLP_14180), and LruB (LIMLP_14170)) [10]. Therefore, the H_2_O_2_-induced response overlaps to some extent with other stress responses. In fact, the accumulation of oxidatively-damaged proteins and DNAs could trigger a general stress response. Consistent with this hypothesis is the change in expression of other stress-related regulators such HrcA, LexA, and RpoE.

Comparing the H_2_O_2_-induced changes in gene expression in the *perR* mutant with that in WT cells indicated that PerR contributes only partially to the H_2_O_2_-induced gene regulation. Among the genes whose expression is markedly changed upon exposure to H_2_O_2_, only *katE*, *ahpC* and *ccp* are under the control of PerR. Surprisingly, even in the absence of PerR, *ahpC* and *ccp* expression are still increased upon exposure to H_2_O_2_, suggesting that additional regulatory mechanisms are involved in the H_2_O_2_-induced gene regulation. In fact, several genes encoding transcriptional regulators, two component systems, and sigma factors had their expression altered by the presence of H_2_O_2_, corroborating the involvement of other regulators in the adaptive response to oxidative stress in pathogenic *Leptospira* (Fig 9B). Moreover, we have identified several ncRNAs that might also influence the expression of the H_2_O_2_-regulated genes. Noticeably, rh859 located downstream *ccp* might participate in the increased expression of this gene, together with the derepression induced by PerR dissociation from DNA in the presence of H_2_O_2_ (Fig 9). Therefore, our study has unveiled the complexity of the regulatory network involved in the leptospiral response to oxidative stress.

In the present study, we have further studied the PerR-mediated gene expression control by showing that PerR binds the upstream region of the *ank-katE* operon, indicating that PerR directly represses this operon. *Leptospira* PerR was shown in this study to bind more than 1 kb upstream *ccp* and 500 bp upstream of LIMLP_04285. Such a binding at a distal site from this ORF promoter regions would be consistent with a control of expression mediated by DNA deformation (such as binding or looping) induced by PerR binding. Such a mechanism was demonstrated with the Fur regulator in *Helicobacter pylori* [39].

Among the ORF that are significantly up-regulated in the presence of H_2_O_2_, catalase and ClpB have been shown to be required for *Leptospira* survival under oxidative stress and virulence [9,37]. In the present study, we have confirmed the essential role of *katE* for the defense against H_2_O_2_, particularly in stationary phase. Furthermore, we have identified new ORFs that participate in *Leptospira* survival in the presence of ROS. Indeed, our findings indicate that AhpC, a TBDT system, the lipoprotein LipL48, and the response regulator VicR are involved in *Leptospira* survival in the presence of a superoxide-generating compound. Interestingly, pathogenic *Leptospira* do not encode any gene homolog to a superoxide dismutase or superoxide reductase, nor they exhibit any SOD activity [40]. This is quite intriguing as it is generally believed that all aerobic bacteria do have a SOD. One fundamental question is to understand the mechanism these pathogenic bacteria use to detoxify superoxide produced endogenously during the respiratory chain or exogenously by phagocytic cells during infection. Our study is the first to identify leptospiral factors in pathogenic *Leptospira* involved in survival in the presence of superoxide-generating compound. AhpC could detoxify H_2_O_2_ produced upon the reduction of superoxide, but the exact function of ExbD, the TBDT, and LipL48 in superoxide detoxification is still unclear. In bacteria, ExbD is part of the inner membrane complex TonB/ExbD/ExbB that uses proton motive force to provide the energy necessary by TonB-dependent transporters for uptake of metal chelates, such as siderophore, or Vitamin B12. The presence of LipL48-encoding ORF in the same operon as the TBDT strongly suggests that these two proteins are functionally linked. This TBDT machinery could be involved in the uptake of metals used by a ROS detoxification enzyme or even acting by themself as ROS scavenger. Indeed, manganese has been shown to scavenge superoxide in *Lactobacillus plantarum* and *Neisseria gonorrhoeae*, independently to any SOD activity [41,42].

In conclusion, the present study has revealed, for the first time, the genome-wide general response to peroxide in pathogenic *Leptospira*, revealing putative biological pathways *Leptospira* have evolved to overcome the deadly effect of ROS. Peroxide-induced response involves detoxifying enzymes, molecular chaperones and DNA repair machineries. We have also uncovered a complex regulatory network of transcriptional regulators, sigma factors, two component systems and non-coding RNAs that could orchestrate together with PerR the peroxide adaptive response. Peroxide-induced response also engages a large number of non-annotated and sometimes *Leptospira*-specific ORFs reflecting our limited knowledge on these bacteria physiology.

## Materials and methods

### Bacterial strains and growth condition

*L*. *interrogans* serovar Manilae strain L495 and transposon mutant strains (see S7 Table for a complete description of the transposon mutants used in this study) were grown aerobically at 30°C in Ellinghausen-McCullough-Johnson-Harris medium (EMJH) [43] with shaking at 100 rpm. It should be noted that EMJH medium contains 180 μM FeSO_4_. Cell growth was followed by measuring the absorbance at 420 nm.

### RNA purification

Virulent *L*. *interrogans* serovar Manilae strain L495 and *perR* mutant M776 with less than three *in vitro* passages were used in this study. Four independent biological replicates of exponentially grown WT and *perR* mutant *L*. *interrogans* strains were incubated in the presence or absence of 10 μM H_2_O_2_ for 30 min at 30°C. WT L495 strain was also incubated in the presence of 1 mM H_2_O_2_ for 60 min at 30°C. Harvested cells were resuspended in 1 ml TRIzol (ThermoFisher Scientific) and stored at -80°C. Nucleic Acids were extracted with chloroform and precipitated with isopropanol as described elsewhere [44]. Contaminating genomic DNA was removed by DNAse treatment using the RNAse-free Turbo DNA-free turbo kit (ThermoFisher Scientific) as described by the manufacturer. The integrity of RNAs (RIN > 7.6) was verified by the Agilent Bioanalyzer RNA NanoChips (Agilent technologies, Wilmington, DE).

### RNA Sequencing

rRNA were depleted from 0.5 μg of total RNA using the Ribo-Zero rRNA Removal Kit (Bacteria) from Illumina. Sequencing libraries were constructed using the TruSeq Stranded mRNA Sample preparation kit (20020595) following the manufacturer’s instructions (Illumina). The directional libraries were controlled on Bioanalyzer DNA1000 Chips (Agilent Technologies) and concentrations measured with the Qubit dsDNA HS Assay Kit (ThermoFisher). Sequences of 65 bases were generated on the Illumina Hiseq 2500 sequencer.

Bioinformatics analyses were performed using the RNA-seq pipeline from Sequana [45]. Reads were cleaned of adapter sequences and low-quality sequences using cutadapt version 1.11 [46]. Only sequences at least 25 nt in length were considered for further analysis. Bowtie version 1.2.2 [47], with default parameters, was used for alignment on the reference genome (*L*. *interrogans* serovar Manilae strain UP-MMC-NIID LP, from MicroScope Platform, https://mage.genoscope.cns.fr/microscope/home/index.php). Genes were counted using featureCounts version 1.4.6-p3 [48] from Subreads package (parameters: -t gene -g locus_tag -s 1).

Count data were analyzed using R version 3.5.1 [49] and the Bioconductor package DESeq2 version 1.20.0 [50]. The normalization and dispersion estimation were performed with DESeq2 using the default parameters and statistical tests for differential expression were performed applying the independent filtering algorithm. Differential expressions were expressed as logarithm to base 2 of fold change (Log_2_FC). A generalized linear model including the replicate effect as blocking factor was set in order to test for the differential expression between *Leptospira* samples. Raw p-values were adjusted for multiple testing according to the Benjamini and Hochberg (BH) procedure [51] and genes with an adjusted p-value lower than 0.005 and a Log_2_FC higher than 1 or lower than -1 were considered differentially expressed. The Fisher statistical test was used for the COG (Clusters of Orthologous Groups) classification. Heat maps were generated using the Galaxy platform (https://usegalaxy.eu). The data have been deposited in NCBI's Gene Expression Omnibus and are accessible through GEO Series accession number GSE140019 (https://www.ncbi.nlm.nih.gov/geo/query/acc.cgi?acc=GSE140019).

### Quantitative RT-PCR experiments

cDNA synthesis was performed with the cDNA synthesis kit (Biorad) according to the manufacturer’s recommendation. Quantitative PCR was conducted with the SsoFast EvaGreen Supermix (Biorad) as previously described [9,15]. Gene expression was measured with primers described in S8 Table using *flaB* (LIMLP_09410) as a reference gene.

### Non-coding RNA identification

Sequencing data from the *Leptospira* WT and *perR* mutant strains incubated in the absence or presence of H_2_O_2_ were processed with Trimmomatic [52] to remove low-quality bases and adapter contaminations. BWA mem (version 0.7.12) was used to discard the reads matching *Leptospira* rRNA, tRNA or polyA sequences and to assign the resulting reads to *Leptospira* replicons. Then Rockhopper [53] was used to re-align reads corresponding to separate replicons and to assemble transcripts models. The output was filtered to retain all transcripts longer than 50 nucleotides not overlapping within 10 nucleotides with NCBI annotated genes on the same orientation, and showing a minimum Rockhopper raw count value of 50 in at least two isolates. This high-quality set of 778 new sRNAs was subjected to differential expression analysis with Rockhopper, adopting a Benjamini-Hochberg adjusted P-value threshold of 0.01. For each non-coding RNAs, putative function was identified by BLAST using the Rfam database [54].

### ChIP-qPCR

Chromatin immunoprecipitation was performed by incubating exponentially growing *Leptospira* WT or *perR1* mutant cells 40 min with 1% formaldehyde at 30°C. The reaction was stopped by the addition of 400 mM glycine. Cells were then washed with TBS buffer and resuspended in buffer A (50 mM HEPES-KOH pH7.5, 150 mM NaCl, 1 mM EDTA, 1% Triton X-100) containing a protease inhibitor cocktail. Cells were sonicated 7 cycles of 15 min. and centrifuged. The supernatant was incubated 3 hours at 4°C with 50 μl of washed Dynabead Pan rabbit IgG. The samples were incubated in the absence or in the presence of anti-PerR serum (at a dilution of 1:750) for 2 hours at 4°C. The samples were successively washed with buffer A containing 500 mM NaCl, with buffer B (10 mM Tris-HCl pH8, 1 mM EDTA, 0.1% Nonidet-P40, 0.5% sodium deoxycholate) and with buffer C (10 mM Tris-HCl pH7.5, 1 mM EDTA). The elution was performed with 100 μl of elution buffer (50 mM Tris-HCl pH7.5, 10 mM EDTA, 1% SDS, 150 mM NaCl, 0.5% Triton X-100) upon an ON incubation at 37°C. An incubation with protease K (2 hours at 65°C) allowed elimination of proteins and DNA fragments were purified. DNA fragments were amplified by qPCR using the indicated primers (see S8 Table) and the SsoFast EvaGreen Supermix (Biorad). Results were normalized by the Fold enrichment method (signal over background) calculated using the following formula: 2^ΔΔCq where ΔΔCq is Cq(with antibody) - Cq(without antibody).

### Determination of cell viability

*L*. *interrogans* were cultivated in EMJH medium until logarithmic or stationary phase and diluted to ≈ 10^8^/ml. Cells were then incubated in EMJH in the presence or absence of H_2_O_2_ for the indicated time. Rezasurin (Alamar Blue Assay, ThermoFisher Scientific) was added and cells were further incubated for 24h. Viability is assessed by the reduction of blue resazurin into pink resorufin [55]. Plating experiments were performed by diluting treated and non-treated cells in EMJH in the absence of H_2_O_2_ and plating the samples on EMJH agar medium [55]. Colonies were counted after one-month incubation at 30°C.

### Infection experiments

*L*. *interrogans* WT and mutant strains were cultivated in EMJH medium until the exponential phase and counted under a dark-field microscope using a Petroff-Hauser cell. 10^4^ or 10^6^ bacteria (in 0.5 ml) were injected intraperitoneally in groups of 4–8 male 4 weeks-old Syrian Golden hamsters (RjHan:AURA, Janvier Labs). Animals were monitored daily and sacrificed by CO_2_ inhalation when endpoint criteria were met (sign of distress, morbidity).

### Ethics statement

The protocol for animal experimentation was reviewed by the Institut Pasteur (Paris, France), the competent authority, for compliance with the French and European regulations on Animal Welfare and with Public Health Service recommendations. This project has been reviewed and approved (CETEA #2016–0019) by the Institut Pasteur ethic committee for animal experimentation, agreed by the French Ministery of Agriculture.

## Supporting information

S1 Fig Increase of the *ank-katE* operon expression upon exposure to sublethal dose of H_2_O_2_. (A) Schematic representation of the *ank-katE* locus. The DNA fragments amplified by the PCR in (B) are designated with a bar and their corresponding size is indicated in base pairs in parenthesis. The number of nucleotides between different ORFs is indicated in italic. (B) Electrophoresis gels of the PCR-amplified DNA fragments designated in (A) from genomic DNA (gDNA) or from RNA before (RNA) or after (cDNA) a reverse transcriptase reaction. DNA ladder fragment sizes are indicated at left of the gels. (C) Gene expression was measured by RT-qPCR reactions in WT *L*. *interrogans* exposed in the absence (white bars) or presence of 10 μM H_2_O_2_ for 30 min (black bars) or 2h (dashed bars). Data are mean and SD of three independent experiments. ***, p-value<0.0001 by two-way Anova analysis.
(TIF)

S2 Fig Increase of *ahpC* and *ccp* expressions upon exposure to sublethal dose of H_2_O_2_. *L*. *interrogans* WT cells were cultivated until exponential phase and exposed in the absence (white bars) or presence of 10 μM H_2_O_2_ for 30 min (black bars) or for 2h (dashed bars). RNAs were purified and cDNAs were subsequently prepared by reverse transcription. *AhpC* (A), *sufB* (A), LIMLP_02790 (B) and *ccp* (B) expressions were measured by RT-qPCR using *flaB* (LIMLP_09410) as reference gene and the data were normalized with untreated samples. Data are mean and SD of three independent experiments. ***, p-value<0.0001 by two-way Anova analysis.
(TIF)

S3 Fig Increase of the heme biosynthesis gene expression upon exposure to sublethal dose of H_2_O_2_. (A) Schematic representation of the heme cluster locus. The DNA fragments amplified by the PCR in (B) are designated with a bar and their corresponding size is indicated in base pairs in parenthesis. The number of nucleotides between different ORFs is indicated in italic. (B) Electrophoresis gels of the PCR-amplified DNA fragments designated in (A) from genomic DNA (gDNA) or from RNA before (RNA) or after (cDNA) a reverse transcriptase reaction. DNA ladder fragment sizes are indicated at left of the gels. (C) Gene expression was measured by RT-qPCR reactions in WT *L*. *interrogans* exposed in the absence (white bars) or presence of 10 μM H_2_O_2_ for 30 min (black bars) or 2h (dashed bars). Data are mean and SD of three independent experiments. ****, p-value<0.0001; **, p-value<0.005; *, p-value<0.05 by two-way Anova analysis.
(TIF)

S4 Fig Increase of the LIMLP_17860–65 operon expression upon exposure to sublethal dose of H_2_O_2_. (A) Schematic representation of the locus of genes coding for a heme oxygenase and a permease. The DNA fragments amplified by the PCR in (B) are designated with a bar and their corresponding size is indicated in base pairs in parenthesis. The number of nucleotides between different ORFs is indicated in italic. (B) Electrophoresis gels of the PCR-amplified DNA fragments designated in (A) from genomic DNA (gDNA) or from RNA before (RNA) or after (cDNA) a reverse transcriptase reaction. DNA ladder fragment sizes are indicated at left of the gels. (C) Gene expression was measured by RT-qPCR reactions in WT *L*. *interrogans* exposed in the absence (white bars) or presence of 10 μM H_2_O_2_ for 30 min (black bars) or 2h (dashed bars). Data are mean and SD of three independent experiments. *, p-value<0.05 by two-way Anova analysis.
(TIF)

S5 Fig Low binding of PerR with the PerR-controlled peroxidase locus. Chromatin immunoprecipitation was performed on *L*. *interrogans* WT and *perR* (M776) mutant strains in the presence or absence of the anti-PerR antibody. Co-immunoprecipitated DNA fragments located in the *ank-katE* operon locus (A), in the *ccp* locus (B) and in the *ahpC* locus (C) were amplified by qPCR. The location of amplified fragments is indicated below the schematic representation of their respective loci. The number of nucleotides between different ORFs is indicated in italic. Data are represented as fold enrichments.
(TIF)

S6 Fig Operon organization of the PerR-controlled TonB-dependent transport system locus. (A) Schematic representation of the locus of genes coding for a TonB-dependent transport system. The DNA fragments amplified by the PCR in (B) are designated with a bar and their corresponding size is indicated in base pairs in parenthesis. The number of nucleotides between different ORFs is indicated in italic. (B) Electrophoresis gels of the PCR-amplified DNA fragments designated in (A) from genomic DNA (gDNA) or from RNA before (RNA) or after (cDNA) a reverse transcriptase reaction. DNA ladder fragment sizes are indicated at left of the gels.
(TIF)

S7 Fig Low binding of PerR with the PerR-controlled TonB-dependent transport system locus. Chromatin immunoprecipitation was performed on *L*. *interrogans* WT and *perR* (M776) mutant strains in the presence or absence of the anti-PerR antibody. Co-immunoprecipitated DNA fragments located in the locus encoding a TonB-dependent transporter system were amplified by qPCR. The location of amplified fragments is indicated below the schematic representation of the locus. The number of nucleotides between different ORFs is indicated in italic. Data are represented as fold enrichments.
(TIF)

S8 Fig Operon organization of PerR-controlled *vicKR*. (A) Schematic representation of the locus of genes coding for the histidine kinase VicK and the response regulator VicR. The DNA fragments amplified by the PCR in (B) are designated with a bar and their corresponding size is indicated in base pairs in parenthesis. The number of nucleotides between different ORFs is indicated in italic. (B) Electrophoresis gels of the PCR-amplified DNA fragments designated in (A) from genomic DNA (gDNA) or from RNA before (RNA) or after (cDNA) a reverse transcriptase reaction. DNA ladder fragment sizes are indicated at left of the gels.
(TIF)

S1 Table Complete set of ORF expression in *Leptospira interrogans* WT and M776 *perR* mutant upon a 30 min exposure to 10 μM H_2_O_2_. (XLSX)

S2 Table Complete set of *Leptospira interrogans* ORF expression upon 60 min exposure to 1 mM H_2_O_2_. (XLSX)

S3 Table Selected up-regulated genes upon exposure to lethal doses of H_2_O_2_. (DOCX)

S4 Table Selected down-regulated genes upon exposure to lethal doses of H_2_O_2_. (DOCX)

S5 Table Differentially-expressed ncRNAs upon *perR* inactivation and exposure to sublethal doses of H_2_O_2_. (DOCX)

S6 Table Complete set of differentially-expressed ncRNAs in *Leptospira interrogans* WT and M776 *perR* mutant upon exposure to H_2_O_2_. (XLSX)

S7 Table Transposon mutants used in this study. (DOCX)

S8 Table Primers used in this study. (XLSX)

## References

[1] Haake DA, Levett PN. Leptospirosis in humans. Curr Top Microbiol Immunol. 2015;387:65–97. 10.1007/978-3-662-45059-8_5 25388133 PMC4442676

[2] Costa F, Hagan JE, Calcagno J, Kane M, Torgerson P, Martinez-Silveira MS, et al Global Morbidity and Mortality of Leptospirosis: A Systematic Review. PLoS Negl Trop Dis. 2015 9 17;9(9):e0003898–e0003898. 10.1371/journal.pntd.0003898 26379143 PMC4574773

[3] Pijnacker R, Goris MGA, te Wierik MJM, Broens EM, van der Giessen JWB, de Rosa M, et al Marked increase in leptospirosis infections in humans and dogs in the Netherlands, 2014. Eurosurveillance [Internet]. 2016;21(17). Available from: https://www.eurosurveillance.org/content/10.2807/1560-7917.ES.2016.21.17.30211 10.2807/1560-7917.ES.2016.21.17.30211 27168584

[4] Ko AI, Goarant C, Picardeau M. Leptospira: the dawn of the molecular genetics era for an emerging zoonotic pathogen. Nat Rev Microbiol. 2009 10;7(10):736–47. 10.1038/nrmicro2208 19756012 PMC3384523

[5] Picardeau M. Virulence of the zoonotic agent of leptospirosis: still terra incognita? Nat Rev Microbiol. 2017;15(5):297–307. 10.1038/nrmicro.2017.5 28260786

[6] Marangoni A, Accardo S, Aldini R, Guardigli M, Cavrini F, Sambri V, et al Production of reactive oxygen species and expression of inducible nitric oxide synthase in rat isolated Kupffer cells stimulated by Leptospira interrogans and Borrelia burgdorferi. World J Gastroenterol. 2006 5 21;12(19):3077–81. 10.3748/wjg.v12.i19.3077 16718791 PMC4124385

[7] Araújo AM, Reis EAG, Athanazio DA, Ribeiro GS, Hagan JE, Araujo GC, et al Oxidative stress markers correlate with renal dysfunction and thrombocytopenia in severe leptospirosis. Am J Trop Med Hyg. 2014 4;90(4):719–23. 10.4269/ajtmh.13-0667 24493675 PMC3973519

[8] Erdogan HM, Karapehlivan M, Citil M, Atakisi O, Uzlu E, Unver A. Serum sialic acid and oxidative stress parameters changes in cattle with leptospirosis. Veterinary Research Communications. 2008 4 1;32(4):333–9. 10.1007/s11259-008-9036-z 18247150

[9] Eshghi A, Lourdault K, Murray GL, Bartpho T, Sermswan RW, Picardeau M, et al Leptospira interrogans Catalase Is Required for Resistance to H2O2 and for Virulence. Blanke SR, editor. Infect Immun. 2012 11 1;80(11):3892 10.1128/IAI.00466-12 22927050 PMC3486042

[10] Lo M, Murray GL, Khoo CA, Haake DA, Zuerner RL, Adler B. Transcriptional response of Leptospira interrogans to iron limitation and characterization of a PerR homolog. Infect Immun. 2010 11;78(11):4850–9. 10.1128/IAI.00435-10 20805337 PMC2976334

[11] Faulkner MJ, Helmann JD. Peroxide stress elicits adaptive changes in bacterial metal ion homeostasis. Antioxid Redox Signal. 2011 7 1;15(1):175–89. 10.1089/ars.2010.3682 20977351 PMC3110094

[12] Jacquamet L, Traoré D a. K, Ferrer J-L, Proux O, Testemale D, Hazemann J-L, et al Structural characterization of the active form of PerR: insights into the metal-induced activation of PerR and Fur proteins for DNA binding. Mol Microbiol. 2009 7;73(1):20–31. 10.1111/j.1365-2958.2009.06753.x 19508285

[13] Lee J-W, Helmann JD. The PerR transcription factor senses H2O2 by metal-catalysed histidine oxidation. Nature. 2006 3 16;440(7082):363–7. 10.1038/nature04537 16541078

[14] Traoré DAK, El Ghazouani A, Jacquamet L, Borel F, Ferrer J-L, Lascoux D, et al Structural and functional characterization of 2-oxo-histidine in oxidized PerR protein. Nat Chem Biol. 2009 1;5(1):53–9. 10.1038/nchembio.133 19079268

[15] Kebouchi M, Saul F, Taher R, Landier A, Beaudeau B, Dubrac S, et al Structure and function of the Leptospira interrogans peroxide stress regulator (PerR), an atypical PerR devoid of a structural metal-binding site. J Biol Chem. 2018 1 12;293(2):497–509. 10.1074/jbc.M117.804443 29146596 PMC5767856

[16] Caimano MJ, Sivasankaran SK, Allard A, Hurley D, Hokamp K, Grassmann AA, et al A Model System for Studying the Transcriptomic and Physiological Changes Associated with Mammalian Host-Adaptation by Leptospira interrogans Serovar Copenhageni. PLOS Pathogens. 2014 3 13;10(3):e1004004 10.1371/journal.ppat.1004004 24626166 PMC3953431

[17] Satou K, Shimoji M, Tamotsu H, Juan A, Ashimine N, Shinzato M, et al Complete Genome Sequences of Low-Passage Virulent and High-Passage Avirulent Variants of Pathogenic Leptospira interrogans Serovar Manilae Strain UP-MMC-NIID, Originally Isolated from a Patient with Severe Leptospirosis, Determined Using PacBio Single-Molecule Real-Time Technology. Genome Announc. 2015 8 13;3(4):e00882–15. 10.1128/genomeA.00882-15 26272567 PMC4536678

[18] Faine S. Catalase activity in pathogenic Leptospira. J Gen Microbiol. 1960 2;22:1–9. 10.1099/00221287-22-1-1 13821327

[19] Rao PJ, Larson A D AD, Cox C D CD. Catalase activity in Leptospira. J Bacteriol. 1964 10;88(4):1045–8. 14219017 10.1128/jb.88.4.1045-1048.1964 PMC314852

[20] Flint A, Stintzi A. Cj1386, an atypical hemin-binding protein, mediates hemin trafficking to KatA in Campylobacter jejuni. J Bacteriol. 2015 3;197(5):1002–11. 10.1128/JB.02346-14 25548249 PMC4325098

[21] Howell ML, Alsabbagh E, Ma JF, Ochsner UA, Klotz MG, Beveridge TJ, et al AnkB, a periplasmic ankyrin-like protein in Pseudomonas aeruginosa, is required for optimal catalase B (KatB) activity and resistance to hydrogen peroxide. J Bacteriol. 2000 8;182(16):4545–56. 10.1128/jb.182.16.4545-4556.2000 10913088 PMC94626

[22] Arias DG, Reinoso A, Sasoni N, Hartman MD, Iglesias AA, Guerrero SA. Kinetic and structural characterization of a typical two-cysteine peroxiredoxin from Leptospira interrogans exhibiting redox sensitivity. Free Radic Biol Med. 2014 12;77:30–40. 10.1016/j.freeradbiomed.2014.08.014 25236736

[23] Jensen LMR, Sanishvili R, Davidson VL, Wilmot CM. In crystallo posttranslational modification within a MauG/pre-methylamine dehydrogenase complex. Science. 2010 3 12;327(5971):1392–4. 10.1126/science.1182492 20223990 PMC2878131

[24] Guégan R, Camadro J-M, Saint Girons I, Picardeau M. Leptospira spp. possess a complete haem biosynthetic pathway and are able to use exogenous haem sources. Mol Microbiol. 2003 8;49(3):745–54. 10.1046/j.1365-2958.2003.03589.x 12864856

[25] Zhukova A, Fernandes LG, Hugon P, Pappas CJ, Sismeiro O, Coppée J-Y, et al Genome-Wide Transcriptional Start Site Mapping and sRNA Identification in the Pathogen Leptospira interrogans. Front Cell Infect Microbiol. 2017;7:10 10.3389/fcimb.2017.00010 28154810 PMC5243855

[26] Verma A, Kumar P, Babb K, Timoney JF, Stevenson B. Cross-reactivity of antibodies against leptospiral recurrent uveitis-associated proteins A and B (LruA and LruB) with eye proteins. PLoS Negl Trop Dis. 2010 8 3;4(8):e778 10.1371/journal.pntd.0000778 20689825 PMC2914785

[27] Sasoni N, Iglesias AA, Guerrero SA, Arias DG. Functional thioredoxin reductase from pathogenic and free-living Leptospira spp. Free Radic Biol Med. 2016;97:1–13. 10.1016/j.freeradbiomed.2016.05.008 27178006

[28] Fonseca LS, da Silva JB, Milanez JS, Monteiro-Vitorello CB, Momo L, de Morais ZM, et al Leptospira interrogans serovar copenhageni harbors two lexA genes involved in SOS response. PLoS ONE. 2013;8(10):e76419 10.1371/journal.pone.0076419 24098496 PMC3789691

[29] Schons-Fonseca L, da Silva JB, Milanez JS, Domingos RH, Smith JL, Nakaya HI, et al Analysis of LexA binding sites and transcriptomics in response to genotoxic stress in Leptospira interrogans. Nucleic Acids Res. 2016 2 18;44(3):1179–91. 10.1093/nar/gkv1536 26762976 PMC4756842

[30] Qin J-H, Zhang Q, Zhang Z-M, Zhong Y, Yang Y, Hu B-Y, et al Identification of a novel prophage-like gene cluster actively expressed in both virulent and avirulent strains of Leptospira interrogans serovar Lai. Infect Immun. 2008 6;76(6):2411–9. 10.1128/IAI.01730-07 18362131 PMC2423081

[31] Bourhy P, Louvel H, Saint Girons I, Picardeau M. Random Insertional Mutagenesis of Leptospira interrogans, the Agent of Leptospirosis, Using a mariner Transposon. J Bacteriol. 2005 5 1;187(9):3255–8. 10.1128/JB.187.9.3255-3258.2005 15838053 PMC1082815

[32] Bol DK, Yasbin RE. Analysis of the dual regulatory mechanisms controlling expression of the vegetative catalase gene of Bacillus subtilis. J Bacteriol. 1994 11;176(21):6744–8. 10.1128/jb.176.21.6744-6748.1994 7961428 PMC197032

[33] Visick JE, Clarke S. RpoS- and OxyR-independent induction of HPI catalase at stationary phase in Escherichia coli and identification of rpoS mutations in common laboratory strains. J Bacteriol. 1997 7;179(13):4158–63. 10.1128/jb.179.13.4158-4163.1997 9209028 PMC179234

[34] Khademian M, Imlay JA. Escherichia coli cytochrome c peroxidase is a respiratory oxidase that enables the use of hydrogen peroxide as a terminal electron acceptor. Proc Natl Acad Sci U S A. 2017 8 15;114(33):E6922–31. 10.1073/pnas.1701587114 28696311 PMC5565418

[35] Lo M, Bulach DM, Powell DR, Haake DA, Matsunaga J, Paustian ML, et al Effects of temperature on gene expression patterns in Leptospira interrogans serovar Lai as assessed by whole-genome microarrays. Infect Immun. 2006 10;74(10):5848–59. 10.1128/IAI.00755-06 16988264 PMC1594916

[36] Lo M, Cordwell SJ, Bulach DM, Adler B. Comparative transcriptional and translational analysis of leptospiral outer membrane protein expression in response to temperature. PLoS Negl Trop Dis. 2009 12 8;3(12):e560–e560. 10.1371/journal.pntd.0000560 19997626 PMC2780356

[37] Lourdault K, Cerqueira GM, Wunder EA, Picardeau M. Inactivation of clpB in the pathogen Leptospira interrogans reduces virulence and resistance to stress conditions. Infect Immun. 2011 9;79(9):3711–7. 10.1128/IAI.05168-11 21730091 PMC3165490

[38] Matsunaga J, Lo M, Bulach DM, Zuerner RL, Adler B, Haake DA. Response of Leptospira interrogans to physiologic osmolarity: relevance in signaling the environment-to-host transition. Infect Immun. 2007 6;75(6):2864–74. 10.1128/IAI.01619-06 17371863 PMC1932867

[39] Roncarati D, Pelliciari S, Doniselli N, Maggi S, Vannini A, Valzania L, et al Metal-responsive promoter DNA compaction by the ferric uptake regulator. Nature Communications. 2016 8 25;7(1):12593. 10.1038/ncomms12593 PMC5007355 27558202

[40] Austin FE, Barbieri JT, Corin RE, Grigas KE, Cox CD. Distribution of superoxide dismutase, catalase, and peroxidase activities among Treponema pallidum and other spirochetes. Infect Immun. 1981 8;33(2):372–9. 10.1128/IAI.33.2.372-379.1981 7024127 PMC350708

[41] Archibald FS, Fridovich I. The scavenging of superoxide radical by manganous complexes: In vitro. Archives of Biochemistry and Biophysics. 1982 4 1;214(2):452–63. 10.1016/0003-9861(82)90049-2 6284026

[42] Tseng H-J, Srikhanta Y, McEwan AG, Jennings MP. Accumulation of manganese in Neisseria gonorrhoeae correlates with resistance to oxidative killing by superoxide anion and is independent of superoxide dismutase activity. Molecular Microbiology. 2001 6 1;40(5):1175–86. 10.1046/j.1365-2958.2001.02460.x 11401721

[43] Ellinghausen HC, Mccullough WG. Nutrition of Leptospira pomona and growth of 13 other serotypes: a serum-free medium employing oleic albumin complex. Am J Vet Res. 1965 1;26:39–44. 14266933

[44] Zavala-Alvarado C, Benaroudj N. The Single-Step Method of RNA Purification Applied to Leptospira. Methods Mol Biol. 2020;2134:41–51. 10.1007/978-1-0716-0459-5_5 32632858

[45] Cokelaer T, Desvillechabrol D, Legendre R, Cardon M. ‘Sequana’: a Set of Snakemake NGS pipelines. The Journal of Open Source Software. 2(16).

[46] Martin M. Cutadapt removes adapter sequences from high-throughput sequencing reads. EMBnet.journal; Vol 17, No 1: Next Generation Sequencing Data AnalysisDO—1014806/ej171200 [Internet]. 2011 May 2; Available from: http://journal.embnet.org/index.php/embnetjournal/article/view/200

[47] Langmead B, Trapnell C, Pop M, Salzberg SL. Ultrafast and memory-efficient alignment of short DNA sequences to the human genome. Genome Biology. 2009 3 4;10(3):R25 10.1186/gb-2009-10-3-r25 19261174 PMC2690996

[48] Liao Y, Smyth GK, Shi W. featureCounts: an efficient general purpose program for assigning sequence reads to genomic features. Bioinformatics. 2013 11 13;30(7):923–30. 10.1093/bioinformatics/btt656 24227677

[49] R core Team. R, a language and environment for statistcial computing [Internet]. 2016. Available from: https://www.gbif.org/en/tool/81287/r-a-language-and-environment-for-statistical-computing

[50] Love MI, Huber W, Anders S. Moderated estimation of fold change and dispersion for RNA-seq data with DESeq2. Genome Biology. 2014 12 5;15(12):550 10.1186/s13059-014-0550-8 25516281 PMC4302049

[51] Benjamini Y, Hochberg Y. Controlling the False Discovery Rate: A Practical and Powerful Approach to Multiple Testing. Journal of the Royal Statistical Society Series B (Methodological). 1995;57(1):289–300.

[52] Bolger AM, Lohse M, Usadel B. Trimmomatic: a flexible trimmer for Illumina sequence data. Bioinformatics. 2014 8 1;30(15):2114–20. 10.1093/bioinformatics/btu170 24695404 PMC4103590

[53] McClure R, Balasubramanian D, Sun Y, Bobrovskyy M, Sumby P, Genco CA, et al Computational analysis of bacterial RNA-Seq data. Nucleic Acids Res. 2013 8;41(14):e140–e140. 10.1093/nar/gkt444 23716638 PMC3737546

[54] Kalvari I, Argasinska J, Quinones-Olvera N, Nawrocki EP, Rivas E, Eddy SR, et al Rfam 13.0: shifting to a genome-centric resource for non-coding RNA families. Nucleic Acids Research. 2017 11 3;46(D1):D335–42. 10.1093/nar/gkx1038 PMC5753348 29112718

[55] Mouville C, Benaroudj N. Survival Tests for Leptospira spp. Methods Mol Biol. 2020;2134:215–28. 10.1007/978-1-0716-0459-5_20 32632873

