# Supplementary figures and images for: The transcriptional response of pathogenic Leptospira to peroxide reveals new defenses against infection-related oxidative stress
Source: PLoS Pathog. 2020 Oct 6;16(10):e1008904. doi: 10.1371/journal.ppat.1008904 (PMC7567364; doi:10.1371/journal.ppat.1008904)

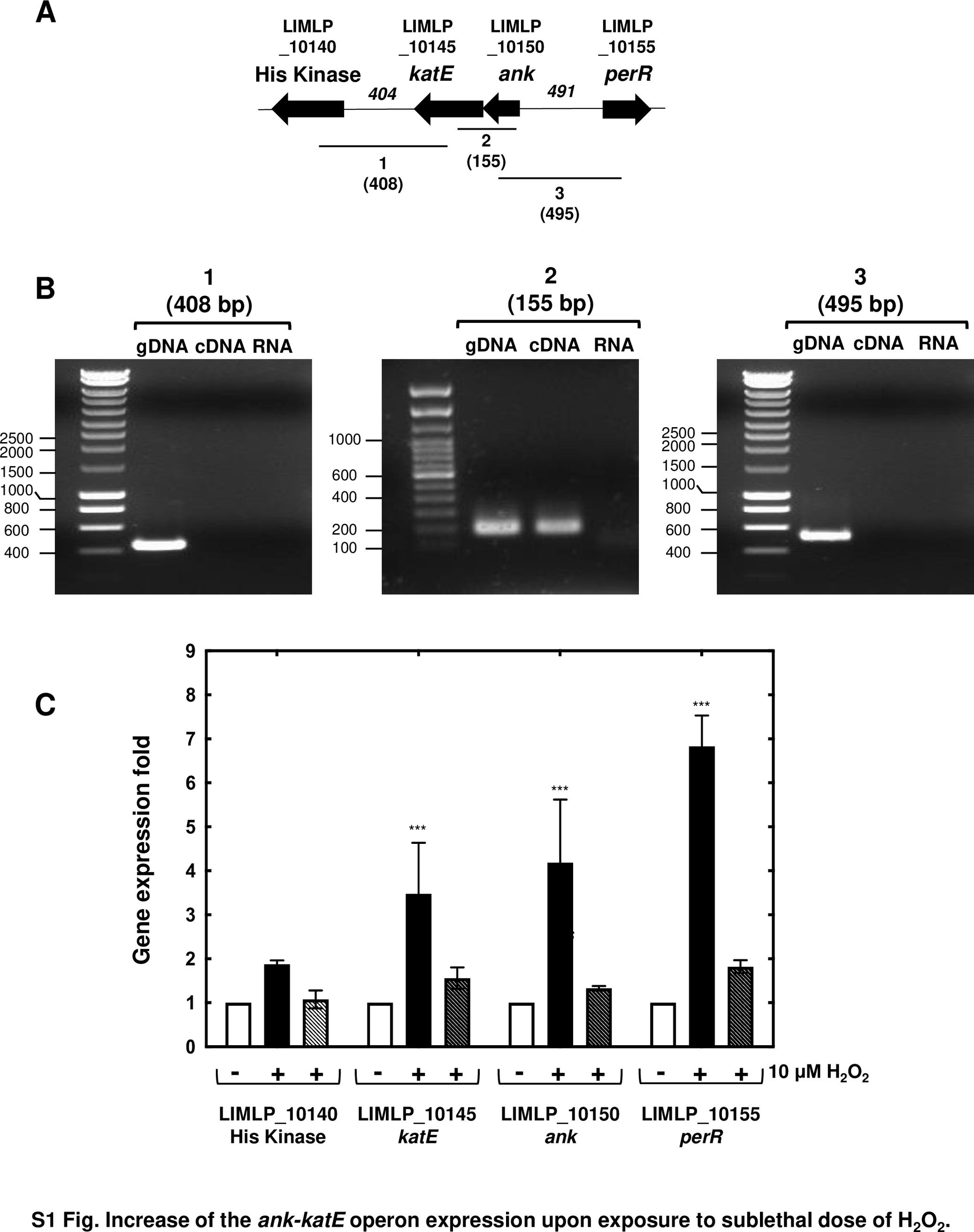

Supplement: S1 Fig — (A) Schematic representation of the ank-katE locus. The DNA fragments amplified by the PCR in (B) are designated with a bar and their corresponding size is indicated in base pairs in parenthesis. The number of nucleotides between different ORFs is indicated in italic. (B) Electrophoresis gels of the PCR-amplified DNA fragments designated in (A) from genomic DNA (gDNA) or from RNA before (RNA) or after (cDNA) a reverse transcriptase reaction. DNA ladder fragment sizes are indicated at left of the gels. (C) Gene expression was measured by RT-qPCR reactions in WT L. interrogans exposed in the absence (white bars) or presence of 10 μM H2O2 for 30 min (black bars) or 2h (dashed bars). Data are mean and SD of three independent experiments. ***, p-value<0.0001 by two-way Anova analysis. (TIF) [file ppat.1008904.s001.tif]

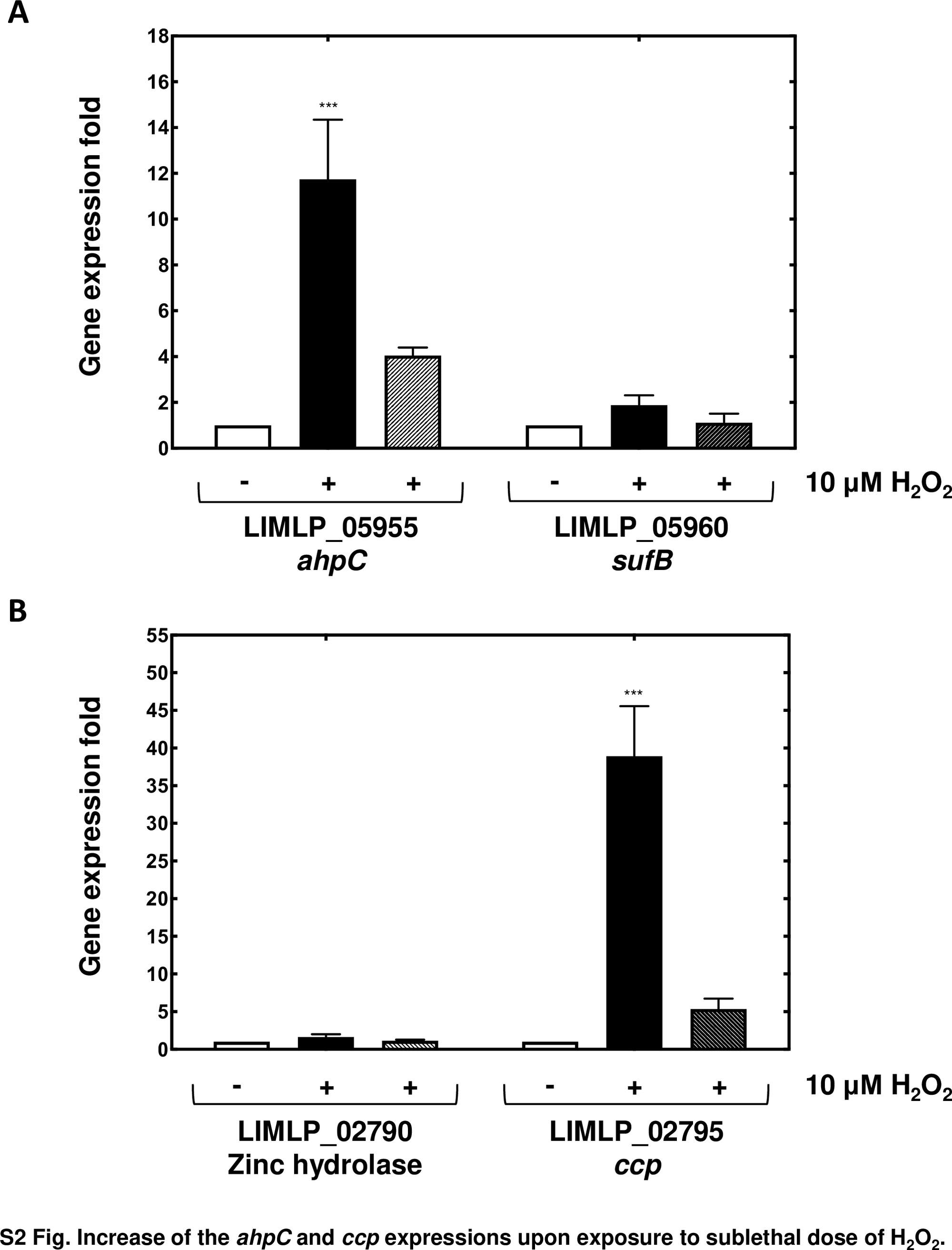

Supplement: S2 Fig — L. interrogans WT cells were cultivated until exponential phase and exposed in the absence (white bars) or presence of 10 μM H2O2 for 30 min (black bars) or for 2h (dashed bars). RNAs were purified and cDNAs were subsequently prepared by reverse transcription. AhpC (A), sufB (A), LIMLP_02790 (B) and ccp (B) expressions were measured by RT-qPCR using flaB (LIMLP_09410) as reference gene and the data were normalized with untreated samples. Data are mean and SD of three independent experiments. ***, p-value<0.0001 by two-way Anova analysis. (TIF) [file ppat.1008904.s002.tif]

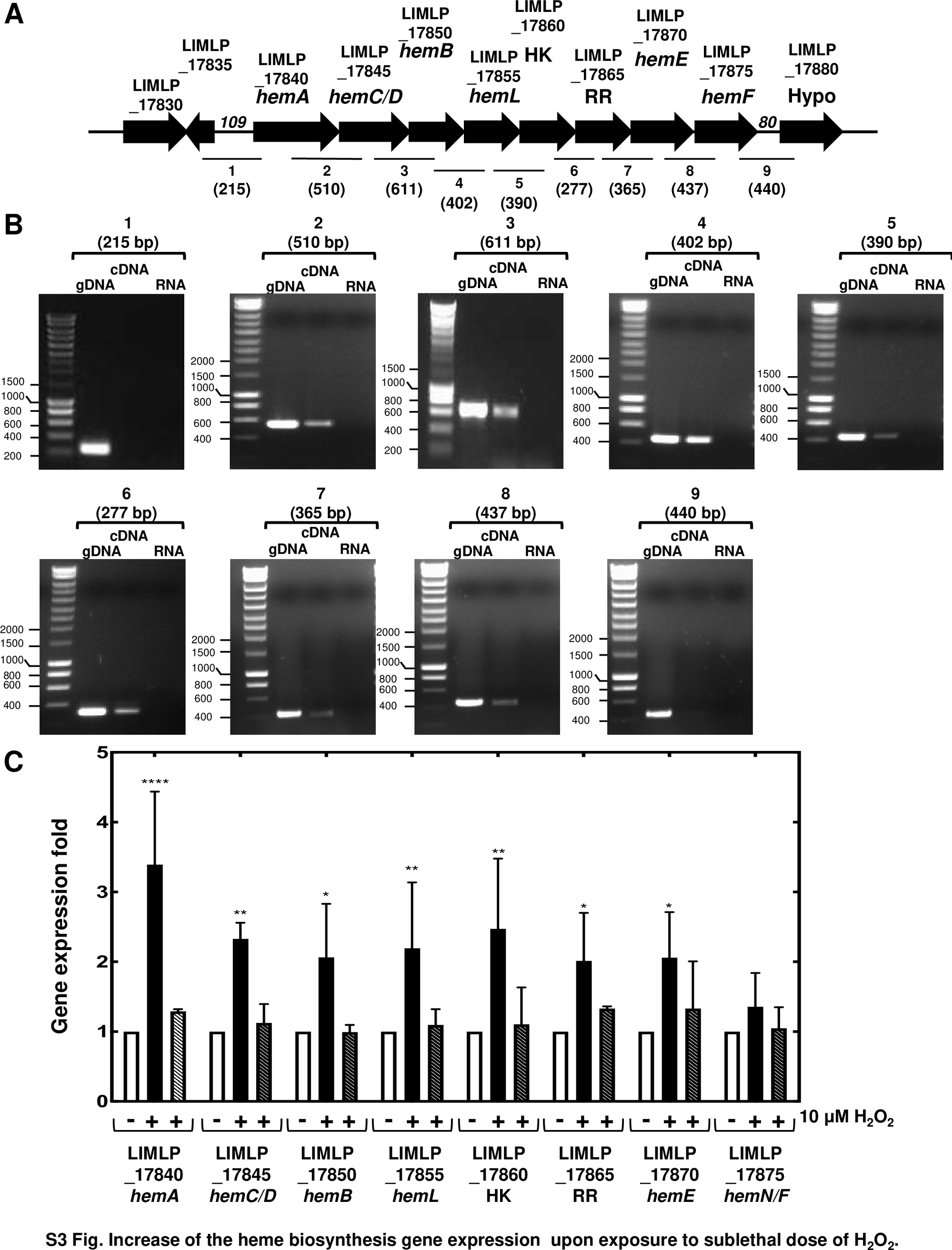

Supplement: S3 Fig — (A) Schematic representation of the heme cluster locus. The DNA fragments amplified by the PCR in (B) are designated with a bar and their corresponding size is indicated in base pairs in parenthesis. The number of nucleotides between different ORFs is indicated in italic. (B) Electrophoresis gels of the PCR-amplified DNA fragments designated in (A) from genomic DNA (gDNA) or from RNA before (RNA) or after (cDNA) a reverse transcriptase reaction. DNA ladder fragment sizes are indicated at left of the gels. (C) Gene expression was measured by RT-qPCR reactions in WT L. interrogans exposed in the absence (white bars) or presence of 10 μM H2O2 for 30 min (black bars) or 2h (dashed bars). Data are mean and SD of three independent experiments. ****, p-value<0.0001; **, p-value<0.005; *, p-value<0.05 by two-way Anova analysis. (TIF) [file ppat.1008904.s003.tif]

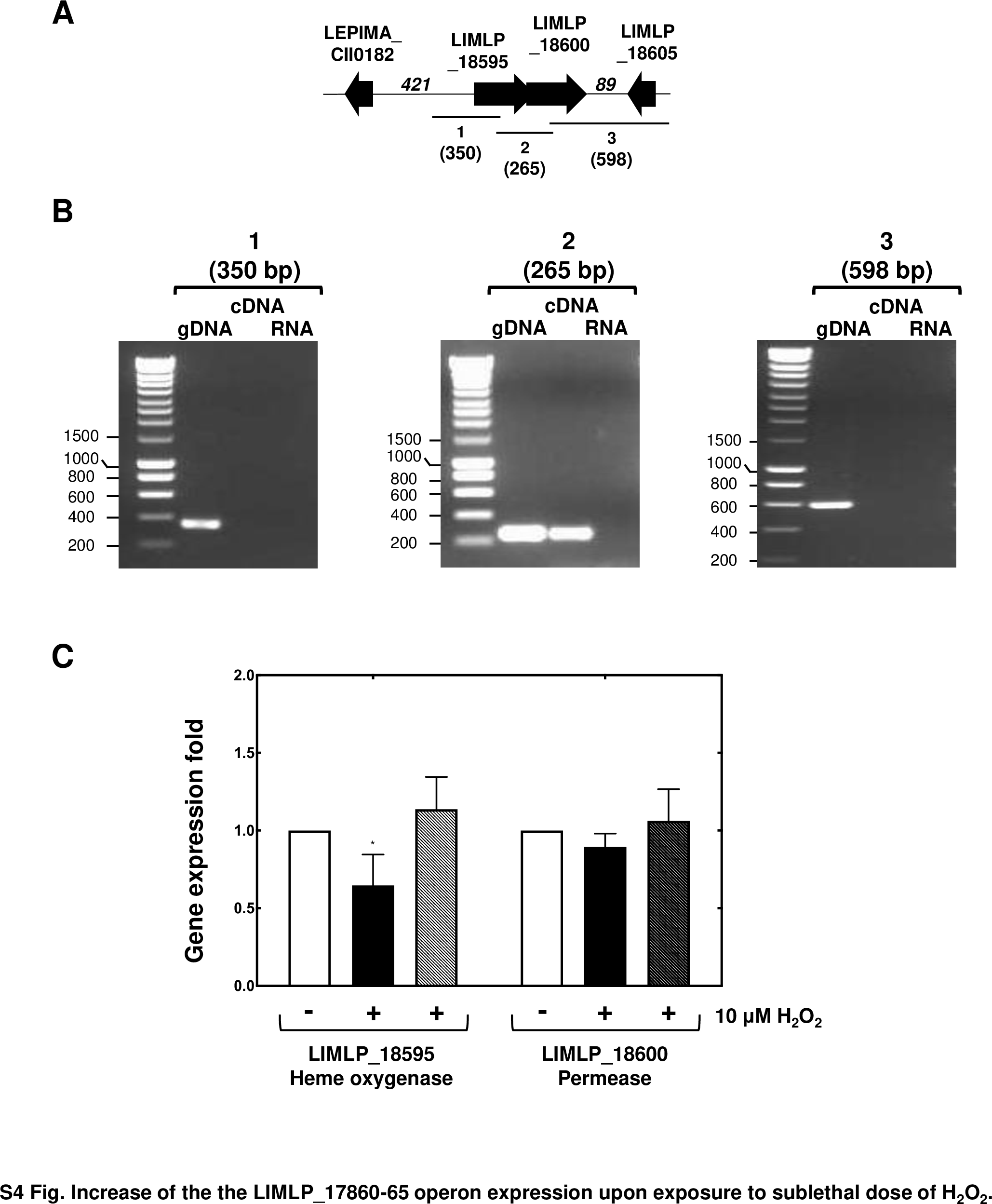

Supplement: S4 Fig — (A) Schematic representation of the locus of genes coding for a heme oxygenase and a permease. The DNA fragments amplified by the PCR in (B) are designated with a bar and their corresponding size is indicated in base pairs in parenthesis. The number of nucleotides between different ORFs is indicated in italic. (B) Electrophoresis gels of the PCR-amplified DNA fragments designated in (A) from genomic DNA (gDNA) or from RNA before (RNA) or after (cDNA) a reverse transcriptase reaction. DNA ladder fragment sizes are indicated at left of the gels. (C) Gene expression was measured by RT-qPCR reactions in WT L. interrogans exposed in the absence (white bars) or presence of 10 μM H2O2 for 30 min (black bars) or 2h (dashed bars). Data are mean and SD of three independent experiments. *, p-value<0.05 by two-way Anova analysis. (TIF) [file ppat.1008904.s004.tif]

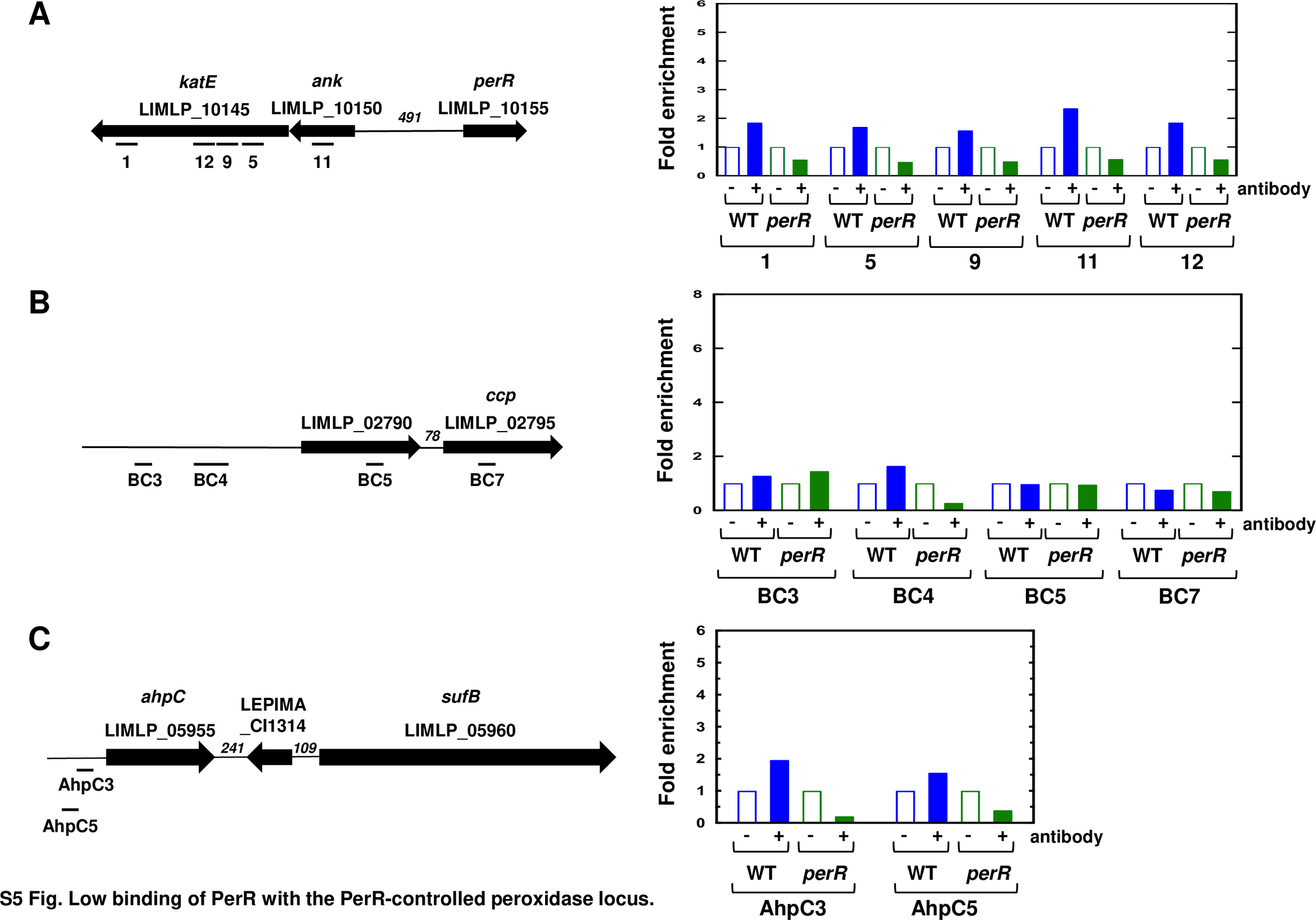

Supplement: S5 Fig — Chromatin immunoprecipitation was performed on L. interrogans WT and perR (M776) mutant strains in the presence or absence of the anti-PerR antibody. Co-immunoprecipitated DNA fragments located in the ank-katE operon locus (A), in the ccp locus (B) and in the ahpC locus (C) were amplified by qPCR. The location of amplified fragments is indicated below the schematic representation of their respective loci. The number of nucleotides between different ORFs is indicated in italic. Data are represented as fold enrichments. (TIF) [file ppat.1008904.s005.tif]

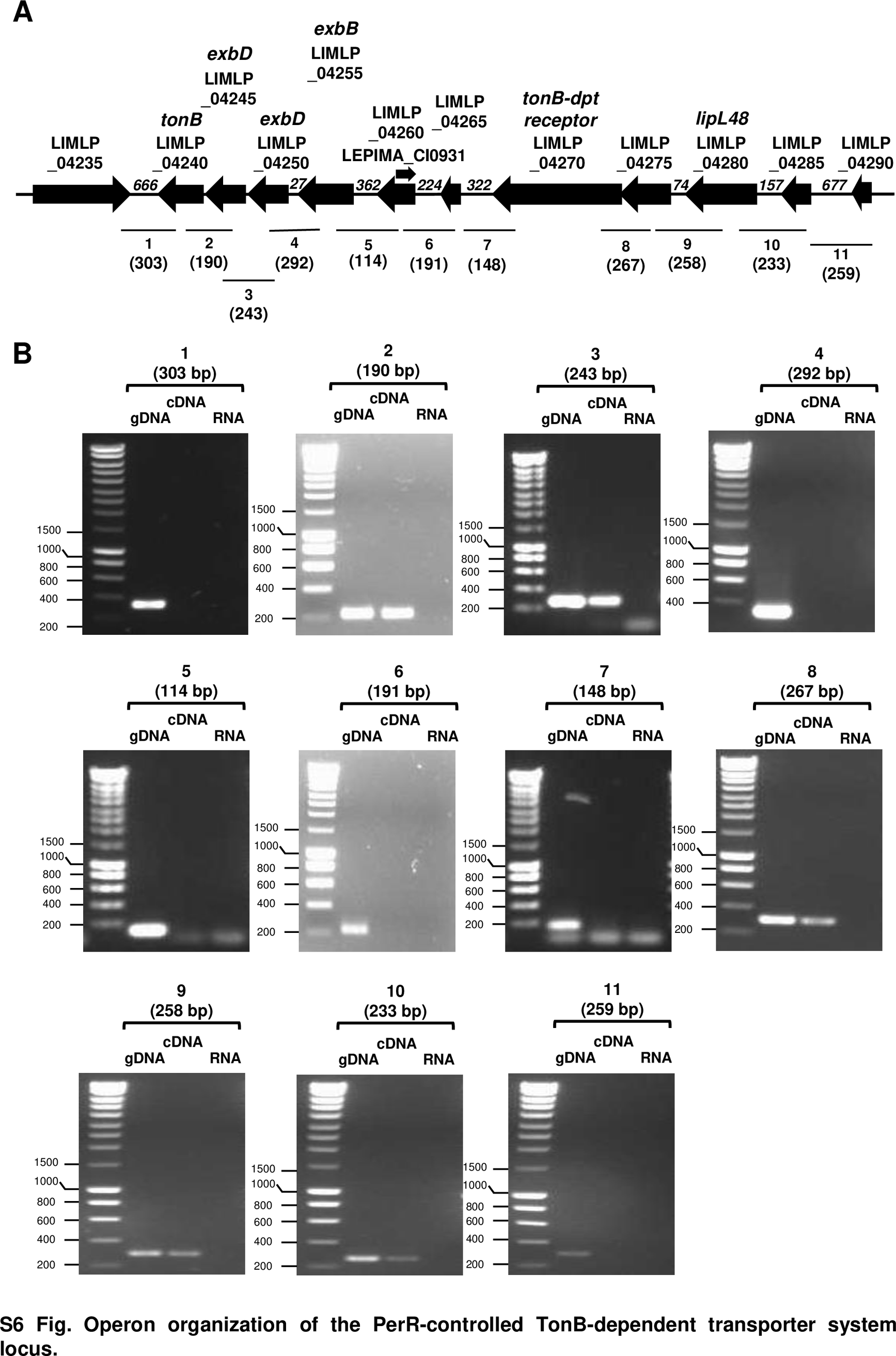

Supplement: S6 Fig — (A) Schematic representation of the locus of genes coding for a TonB-dependent transport system. The DNA fragments amplified by the PCR in (B) are designated with a bar and their corresponding size is indicated in base pairs in parenthesis. The number of nucleotides between different ORFs is indicated in italic. (B) Electrophoresis gels of the PCR-amplified DNA fragments designated in (A) from genomic DNA (gDNA) or from RNA before (RNA) or after (cDNA) a reverse transcriptase reaction. DNA ladder fragment sizes are indicated at left of the gels. (TIF) [file ppat.1008904.s006.tif]

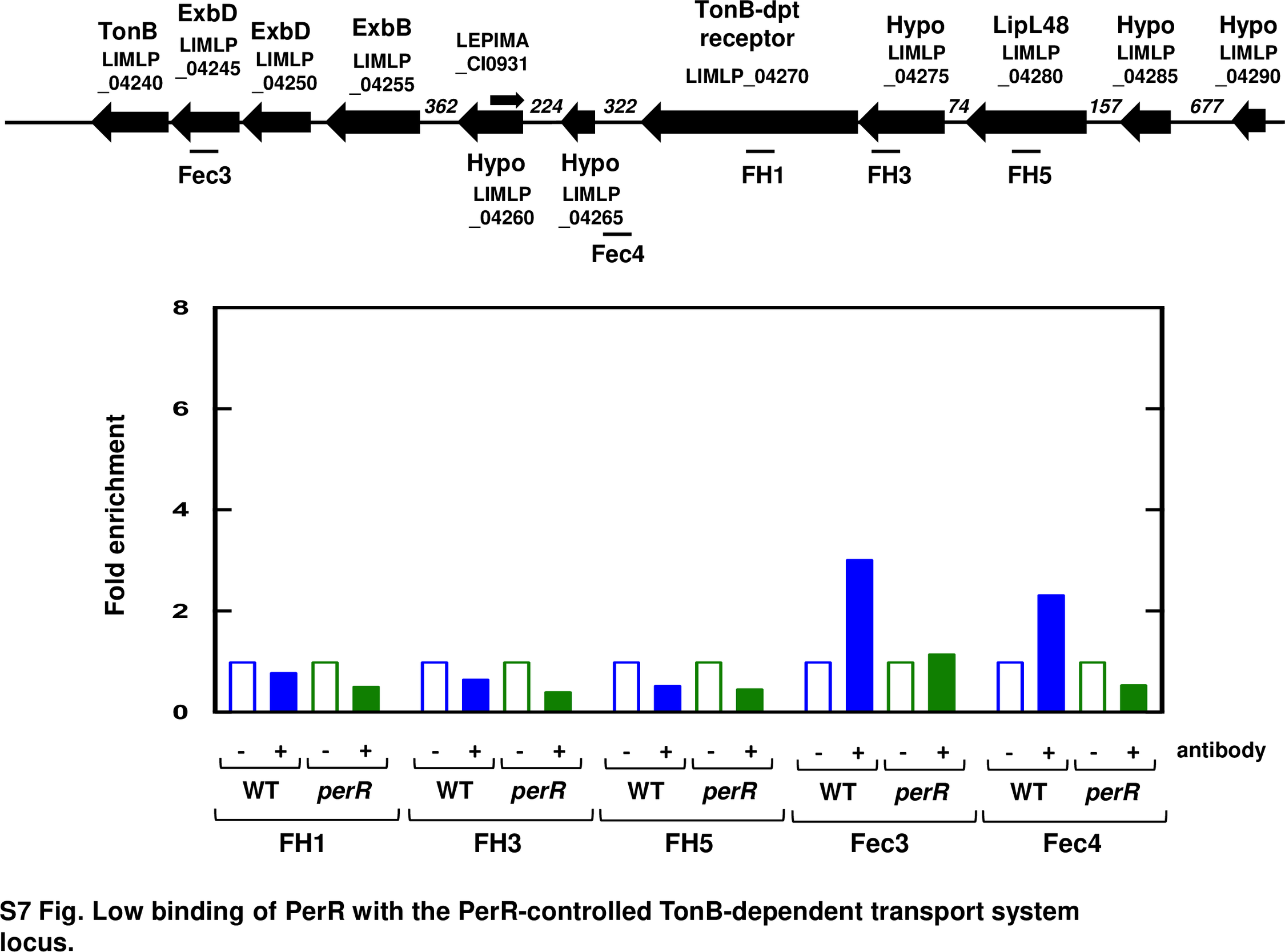

Supplement: S7 Fig — Chromatin immunoprecipitation was performed on L. interrogans WT and perR (M776) mutant strains in the presence or absence of the anti-PerR antibody. Co-immunoprecipitated DNA fragments located in the locus encoding a TonB-dependent transporter system were amplified by qPCR. The location of amplified fragments is indicated below the schematic representation of the locus. The number of nucleotides between different ORFs is indicated in italic. Data are represented as fold enrichments. (TIF) [file ppat.1008904.s007.tif]

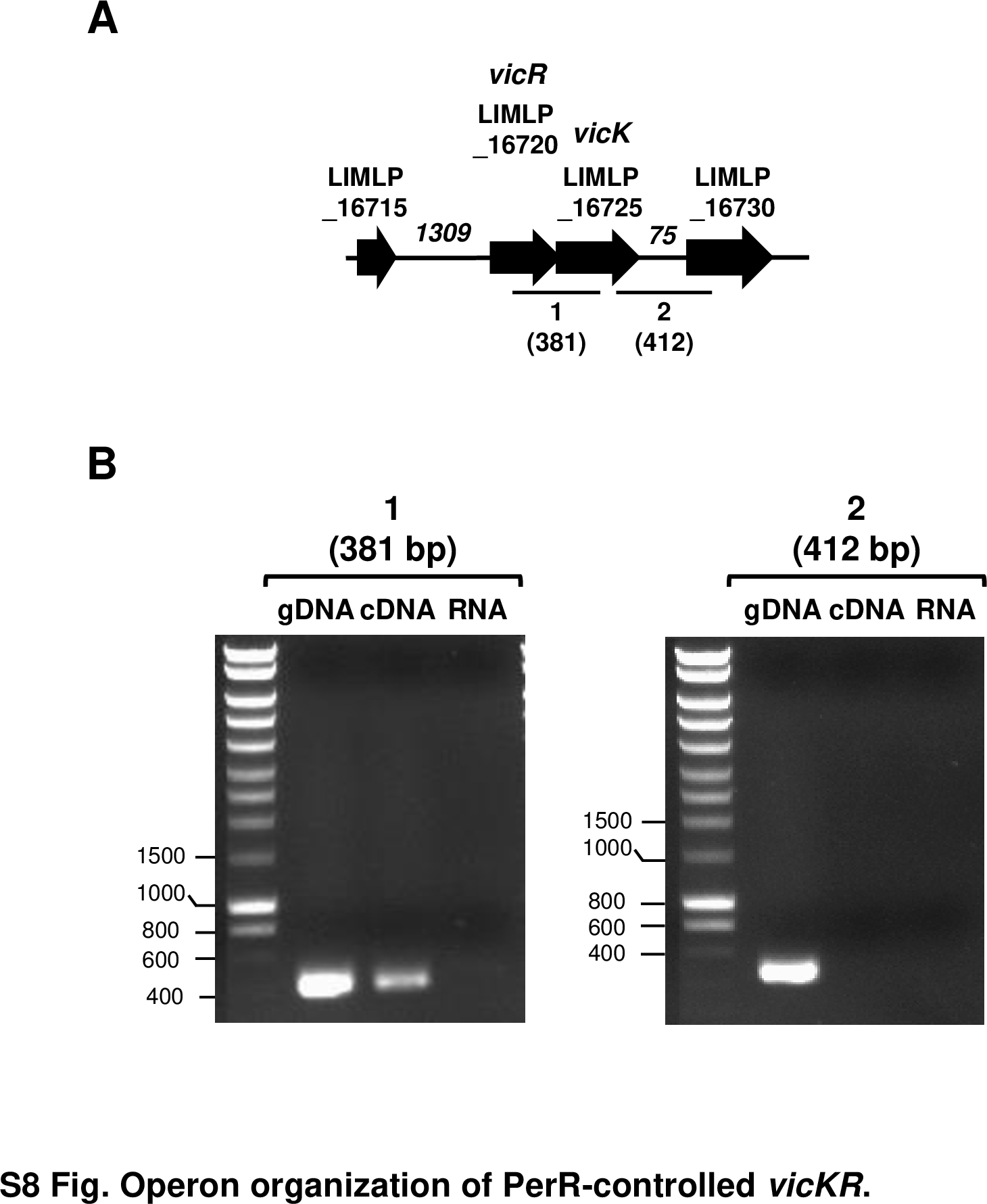

Supplement: S8 Fig — (A) Schematic representation of the locus of genes coding for the histidine kinase VicK and the response regulator VicR. The DNA fragments amplified by the PCR in (B) are designated with a bar and their corresponding size is indicated in base pairs in parenthesis. The number of nucleotides between different ORFs is indicated in italic. (B) Electrophoresis gels of the PCR-amplified DNA fragments designated in (A) from genomic DNA (gDNA) or from RNA before (RNA) or after (cDNA) a reverse transcriptase reaction. DNA ladder fragment sizes are indicated at left of the gels. (TIF) [file ppat.1008904.s008.tif]
