# Supplementary material for: The transcriptional response of pathogenic Leptospira to peroxide reveals new defenses against infection-related oxidative stress
Source: PLoS Pathog. 2020 Oct 6;16(10):e1008904. doi: 10.1371/journal.ppat.1008904 (PMC7567364; doi:10.1371/journal.ppat.1008904)
Supplement: S5 Table — (DOCX) [file ppat.1008904.s013.docx]

| **NC RNA^a^** | **chromosome** | **Log_2_Fc** | **Adjusted p-value** | **Start-End** | **Overlapping ORF** | **Upstream ORF** | **Downstream ORF** |
| --- | --- | --- | --- | --- | --- | --- | --- |
| **WT with H_2_O_2_**  **vs WT^b^** |  |  |  |  |  |  |  |
| rh57^#^ | NZ_CP011933.1 | -2.887 | 1.59e-15 | 23941-24050 | LEPIMA_p0025 | LIMLP_19380 | LIMLP_19385 |
| rh74 | NZ_CP011932.1 | 1.437 | 1.17e-06 | 56523-56736 | NA | LIMLP_18085 | LEPIMA_CII0060 |
| rh206 | NZ_CP011931.1 | -1.867 | 3.90e-03 | 153984-154061 | LIMLP_00690 | LIMLP_00685 | LIMLP_00695 |
| rh392 | NZ_CP011931.1 | -1.667 | 3.29e-02 | 299625-299684 | NA | LIMLP_01350 | LIMLP_01355 |
| rh395 | NZ_CP011931.1 | -1.737 | 6.43e-03 | 301445-301510 | LIMLP_01360 | LIMLP_01355 | LIMLP_01365 |
| rh637^#^ | NZ_CP011931.1 | 1.103 | 1.12e-02 | 501388-501477 | NA | LIMLP_02105 | LIMLP_02110 |
| rh859^#^ | NZ_CP011931.1 | 3.188 | 4.06e-105 | 683752-684074 | NA | LIMLP_02795* | LEPIMA_CI0612 |
| rh895 | NZ_CP011931.1 | 1.388 | 2.19e-05 | 734063-734135 | NA | LIMLP_02950 | LIMLP_02955 |
| rh1064 | NZ_CP011931.1 | 1.190 | 4.75e-03 | 858345-858419 | NA | LIMLP_03585 | LIMLP_03590 |
| rh1827 | NZ_CP011931.1 | 1.836 | 2.73e-09 | 1541659-1541789 | LIMLP_06285 | LEPIMA_CI1388 | LIMLP_06290 |
| rh3130^#^ | NZ_CP011931.1 | 1.917 | 2.17e-11 | 2612368-2612495 | LEPIMA_CI2416 | LIMLP_10975 | LEPIMA_CI2417 |
| rh3164 (RF02543) | NZ_CP011931.1 | -1.807 | 2.87e-03 | 2642149-2642207 | NA | LIMLP_11090 | LIMLP_11095 |
| rh3342 | NZ_CP011931.1 | 1.020 | 1.96e-03 | 2775329-2775511 | NA | LIMLP_11660 | LEPIMA_CI2560 |
| rh3352^#^ | NZ_CP011931.1 | 1.585 | 3.81e-05 | 2787780-2787953 | LIMLP_11710 | LIMLP_11705 | LIMLP_11715 |
| rh3590 | NZ_CP011931.1 | 1.064 | 2.50e-02 | 3005107-3005171 | NA | LIMLP_12625 | LIMLP_12630 |
| rh3999 | NZ_CP011931.1 | 1.902 | 1.02e-13 | 3352754-3352979 | LIMLP_14120 | LIMLP_14115 | LIMLP_14135 |
| rh4413^#^ | NZ_CP011931.1 | 1.209 | 1.60e-06 | 3721204-3721564 | NA | LIMLP_15540 | LIMLP_15545 |
| rh4459^#^ | NZ_CP011931.1 | -1.947 | 2.43e-03 | 3755947-3756010 | NA | LIMLP_15710 | LEPIMA_CI3455 |
| rh4869 | NZ_CP011931.1 | 1.039 | 1.47e-02 | 4095525-4095616 | LIMLP_17200 | LIMLP_17195 | LIMLP_17205 |
| ***perR* vs WT^c^** |  |  |  |  |  |  |  |
| rh106 | NZ_CP011932.1 | 1.248 | 3.27e-04 | 74524-74606 | NA | LIMLP_18155 | LIMLP_18160 |
| rh288 | NZ_CP011931.1 | 3.812 | 0.00 | 197282-197352 | LIMLP_00895 | LIMLP_00890 | LEPIMA_CI0185 |
| rh753^§^ | NZ_CP011931.1 | -1.312 | 4.33e-07 | 602773-602842 | NA | LIMLP_02460 | LIMLP_02465 |
| rh859^#^ | NZ_CP011931.1 | 2.502 | 8.77e-56 | 683752-684074 | NA | LIMLP_02795* | LEPIMA_CI0612 |
| rh1263 | NZ_CP011931.1 | -3.129 | 1.96e-95 | 1032253-1032330 | NA | LIMLP_04265 | LIMLP_04270** |
| rh1990 | NZ_CP011931.1 | -1.585 | 1.50e-09 | 1693954-1694018 | LEPIMA_CI1539 | LIMLP_06885 | LIMLP_06890 |
| rh2234 | NZ_CP011931.1 | -1.120 | 1.68e-03 | 1903652-1903716 | NA | LIMLP_07745 | LIMLP_07750 |
| rh2425 | NZ_CP011931.1 | -1.378 | 1.37e-05 | 2031402-2031466 | NA | LIMLP_08405 | LIMLP_08410 |
| rh2487^#^ | NZ_CP011931.1 | -1.209 | 3.97e-07 | 2083779-2083898 | LIMLP_08585 | LEPIMA_CI1903 | LIMLP_08590 |
| rh2811 | NZ_CP011931.1 | -1.025 | 1.20e-02 | 2345656-2345718 | NA | LIMLP_09820 | LIMLP_09825 |
| rh2901 | NZ_CP011931.1 | 1.073 | 6.05e-05 | 2422195-2422454 | LIMLP_10135 | LIMLP_10130 | LIMLP_10140 |
| rh4480 | NZ_CP011931.1 | 1.000 | 3.77e-02 | 3774801-3774859 | NA | LIMLP_15805 | LIMLP_15810 |
| ***perR* with H_2_O_2_ vs *perR* ^d^** |  |  |  |  |  |  |  |
| rh38^§^ | NZ_CP011932.1 | -2.106 | 3.01e-03 | 32083-32148 | LIMLP_17965 | LIMLP_17960 | LIMLP_17970 |
| rh96 | NZ_CP011932.1 | -2.481 | 7.42e-09 | 68369-68433 | NA | LEPIMA_CII0073 | LIMLP_18135 |
| rh151 | NZ_CP011931.1 | -1.700 | 3.55e-02 | 114296-114360 | LEPIMA_CI0108 | LIMLP_00505 | LIMLP_00510 |
| rh206 | NZ_CP011931.1 | -1.920 | 3.57e-03 | 153984-154061 | LIMLP_00690 | LIMLP_00685 | LIMLP_00695 |
| rh326 | NZ_CP011932.1 | -1.851 | 3.26e-03 | 241122-241244 | NA | LIMLP_18790 | LIMLP_18795 |
| rh347 | NZ_CP011932.1 | -1.898 | 1.13e-02 | 254943-255014 | NA | LIMLP_18845 | LIMLP_18850 |
| rh367 | NZ_CP011932.1 | -3.357 | 1.30e-49 | 268892-268989 | NA | LIMLP_18895 | LIMLP_18900 |
| rh401 | NZ_CP011932.1 | -1.914 | 8.98e-03 | 287311-287372 | NA | LIMLP_18975 | LIMLP_18980 |
| rh479 | NZ_CP011931.1 | -1.485 | 7.93e-03 | 370349-370613 | NA | LIMLP_01630 | LIMLP_01635 |
| rh753^§^ | NZ_CP011931.1 | -3.051 | 3.87e-28 | 602773-602842 | NA | LIMLP_02460 | LIMLP_02465 |
| rh859^#^ | NZ_CP011931.1 | 1.711 | 5.47e-07 | 683752-684074 | NA | LIMLP_02795* | LEPIMA_CI0612 |
| rh928 | NZ_CP011931.1 | -2.115 | 5.16e-04 | 766160-766220 | NA | LIMLP_03050 | LEPIMA_CI0670 |
| rh1026 | NZ_CP011931.1 | -1.665 | 1.95e-03 | 831841-832047 | NA | LIMLP_03455 | LIMLP_03460 |
| rh1069 | NZ_CP011931.1 | -1.547 | 4.77e-02 | 863660-863720 | LEPIMA_CI0783 | LIMLP_03605 | LIMLP_03610 |
| rh1367 | NZ_CP011931.1 | -1.793 | 1.24e-02 | 1118885-1118949 | NA | LIMLP_04620 | LIMLP_04625 |
| rh1388 | NZ_CP011931.1 | -1.765 | 9.03e-03 | 114026-1140088 | LEPIMA_CI01032 | LEPIMA_CI1031 | LIMLP_04690 |
| rh1698 | NZ_CP011931.1 | -1.869 | 7.85e-03 | 1428565-1428634 | LIMLP_05800 | LIMLP_05795 | LIMLP_05805 |
| rh1880^§^ | NZ_CP011931.1 | -2.170 | 9.01e-06 | 1592557-1592621 | LEPIMA_CI1441 | LIMLP_06480 | LEPIMA_CI1442 |
| rh2040 | NZ_CP011931.1 | -1.748 | 4.10e-02 | 1736218-1736282 | LIMLP_07045 | LIMLP_07040 | LIMLP_07050 |
| rh2114^§^ | NZ_CP011931.1 | -2.600 | 3.78e-12 | 1799567-1799634 | NA | LIMLP_07290 | LIMLP_07295 |
| rh2458 (RF02541) | NZ_CP011931.1 | -1.954 | 1.10e-03 | 2056548-2056611 | NA | LEPIMA_CI1884 | LIMLP_08500 |
| rh2850^§^ | NZ_CP011931.1 | -2.087 | 6.10e-05 | 2378010-2378075 | LEPIMA_CI2192 | LIMLP_09945 | LIMLP_09950 |
| rh3164 (RF02543) | NZ_CP011931.1 | -2.585 | 2.95e-07 | 2642149-2642207 | NA | LIMLP_11090 | LIMLP_11095 |
| rh3298 | NZ_CP011931.1 | -2.000 | 1.37e-03 | 2741655-2741711 | NA | LIMLP_11550 | LIMLP_11560 |
| rh3753 | NZ_CP011931.1 | -1.854 | 4.15e-03 | 3152894-3153000 | NA | LIMLP_13250 | LIMLP_13255 |
| rh3894^#^ | NZ_CP011931.1 | -1.938 | 5.78e-03 | 3271638-3271704 | NA | LIMLP_13765 | LIMLP_13770 |
| rh4234 | NZ_CP011931.1 | -2.222 | 5.47e-07 | 3541101-3541196 | NA | LEPIMA_CI3278 | LIMLP_14925 |
| rh4459^#^ | NZ_CP011931.1 | -1.947 | 2.43e-03 | 3755947-3756010 | NA | LIMLP_15710 | LEPIMA_CI3455 |
| rh4680 (RF02541) | NZ_CP011931.1 | -1.466 | 1.37e-02 | 3936966-3937120 | NA | LIMLP_16535 | LIMLP_16540 |
| rh4918^§^ | NZ_CP011931.1 | -2.222 | 6.49e-06 | 4133590-4133654 | NA | LIMLP_17350 | LIMLP_17355 |

**S5 Table: Differentially-expressed ncRNAs upon *perR* inactivation and exposure to sublethal doses of H_2_O_2_.**

^a^ Gene numeration is according to Satou et al. (2015).

^b^ Significantly differentially-expressed ncRNAs upon a 30 min. exposure to 10 μM H_2_O_2_ (Log_2_FC cutoff of ± 1, p-value cutoff of 0.05).

^c^ Significantly differentially-expressed ncRNAs upon *perR* inactivation (M776 mutant) (Log_2_FC cutoff of ± 1, p-value cutoff of 0.05).

^d^ Significantly differentially-expressed ncRNAs in the *perR* mutant (M776 mutant) upon a 30 min. exposure to 10 μM H_2_O_2_ (Log_2_FC cutoff of ± 1, p-value cutoff of 0.05).

^§^ ncRNAs significantly down-regulated upon an 1h exposure to 1 mM H_2_O_2_ (Log_2_FC cutoff of -1, p-value cutoff of 0.05).

^#^ ncRNAs significantly up-regulated upon an 1h exposure to 1 mM H_2_O_2_ (Log_2_FC cutoff of 1, p-value cutoff of 0.05).

* ORFs significantly significantly up-regulated by RNASeq analysis (Log_2_FC cutoff of 1, p-value cutoff of 0.05).

** ORFs significantly significantly down-regulated by RNASeq analysis (Log_2_FC cutoff of -1, p-value cutoff of 0.05).

NA, non-applicable

The Rfam classification of ncRNAs is indicated into parenthesis.
